# Comprehensive Genome Analysis of Cellulose and Xylan-Active CAZymes from the Genus *Paenibacillus*: Special Emphasis on the Novel Xylanolytic *Paenibacillus* sp. LS1

**DOI:** 10.1128/spectrum.05028-22

**Published:** 2023-04-18

**Authors:** Saumashish Mukherjee, Tushar Dilipchand Lodha, Jogi Madhuprakash

**Affiliations:** a Department of Plant Sciences, School of Life Sciences, University of Hyderabad, Gachibowli, Hyderabad, India; b National Centre for Microbial Resource, National Centre for Cell Science, Pune, India; Suranaree University of Technology

**Keywords:** *Paenibacillus* spp., xylan, CAZymes, Genome analysis, qRT-PCR

## Abstract

Xylan is the most abundant hemicellulose in hardwood and graminaceous plants. It is a heteropolysaccharide comprising different moieties appended to the xylose units. Complete degradation of xylan requires an arsenal of xylanolytic enzymes that can remove the substitutions and mediate internal hydrolysis of the xylan backbone. Here, we describe the xylan degradation potential and underlying enzyme machinery of the strain, *Paenibacillus* sp. LS1. The strain LS1 was able to utilize both beechwood and corncob xylan as the sole source of carbon, with the former being the preferred substrate. Genome analysis revealed an extensive xylan-active CAZyme repertoire capable of mediating efficient degradation of the complex polymer. In addition to this, a putative xylooligosaccharide ABC transporter and homologues of the enzymes involved in the xylose isomerase pathway were identified. Further, we have validated the expression of selected xylan-active CAZymes, transporters, and metabolic enzymes during growth of the LS1 on xylan substrates using qRT-PCR. The genome comparison and genomic index (average nucleotide identity [ANI] and digital DNA-DNA hybridization) values revealed that strain LS1 is a novel species of the genus *Paenibacillus*. Lastly, comparative genome analysis of 238 genomes revealed the prevalence of xylan-active CAZymes over cellulose across the *Paenibacillus* genus. Taken together, our results indicate that *Paenibacillus* sp. LS1 is an efficient degrader of xylan polymers, with potential implications in the production of biofuels and other beneficial by-products from lignocellulosic biomass.

**IMPORTANCE** Xylan is the most abundant hemicellulose in the lignocellulosic (plant) biomass that requires cooperative deconstruction by an arsenal of different xylanolytic enzymes to produce xylose and xylooligosaccharides. Microbial (particularly, bacterial) candidates that encode such enzymes are an asset to the biorefineries to mediate efficient and eco-friendly deconstruction of xylan to generate products of value. Although xylan degradation by a few *Paenibacillus* spp. is reported, a complete genus-wide understanding of the said trait is unavailable till date. Through comparative genome analysis, we showed the prevalence of xylan-active CAZymes across *Paenibacillus* spp., therefore making them an attractive option towards efficient xylan degradation. Additionally, we deciphered the xylan degradation potential of the strain *Paenibacillus* sp. LS1 through genome analysis, expression profiling, and biochemical studies. The ability of *Paenibacillus* sp. LS1 to degrade different xylan types obtained from different plant species, emphasizes its potential implication in lignocellulosic biorefineries.

## INTRODUCTION

The world is progressing toward natural and renewable resources for generation of energy (as fuels) and chemicals, with the goal of securing a sustainable carbon bioeconomy for the future (1). Potential resources mainly include nonedible biomass, which can be either plant derived (lignocellulose) or chitin based. Lignocellulose is the most abundant complex polymer on Earth, and it comprises polysaccharides such as cellulose, hemicellulose, and pectin and phenolic complexes such as lignin. It is highly recalcitrant yet a promising, renewable resource for a multitude of biotechnological applications (2, 3). Hemicellulose is the second-most abundant plant polysaccharide after cellulose and represents about 20 to 35% of the lignocellulosic biomass (1, 4), depending on the plant developmental stage, tissue type, and species. Hemicelluloses are adsorbed to the surface of cellulose microfibrils, thereby forming a complex network contributing to the recalcitrance of lignocellulosic biomass (2, 3).

Among the hemicelluloses, xylan is the most abundant type in higher plants, particularly in graminaceous plants and hardwoods. Xylan is a heteropolymer of β-1→4-linked d-xylose (d-xyl) units that are decorated with different substitution groups. The commonly observed substitutions are l-arabinofuranosyl (l-Araf), 4-*O*-methyl glucuronyl (d-MeGlcAp), and acetyl residues at the O-2 and/or O-3 positions. Also, l-Araf moieties sometimes are esterified to ferulate at the O-5 position (5). The different substitutions occur depending on the different plant tissue type, species, and developmental stage. The dicots (hardwood species) have glucuronoxylan in their secondary walls, accounting for up to 20 to 30% of the polysaccharide content. The monocots (graminaceous plants) are composed of glucuronoarabinoxylan, accounting for up to 20 to 40% and 40 to 50% of the polysaccharide content in their primary and secondary walls, respectively (6). Glucuronoxylan and glucuronoarabinoxylan mainly differ in the relative amounts of d-MeGlcAp and l-Araf present in them (5). While acetylation of xylose units is a common phenomenon in the dicot (hardwood) cell walls, the presence of ferulic acid esters attached to arabinofuranosyl residues is common to xylans in graminaceous plants (6). Apart from this, a very low content of glucuronoarabinoxylan (2% in primary and 5 to 15% in secondary walls) is also observed in softwood species such as conifers (6). Taking together the complexity and heterogeneity of the xylan polymer, it is important that efficient degradation requires a suite of enzymes acting in synergy toward the removal of individual substitutions, hydrolysis of the primary polymeric chain, and further breakdown of the generated oligomers to monomeric xylose units.

*Paenibacillus* sp. LS1 was isolated from Lachung, Sikkim, India, and was previously investigated for its chitinolytic potential (7). While investigating its ability to degrade other polysaccharides, the development of a visible zone of clearance on M9 medium supplemented with beechwood xylan (1%) agar (Fig. S1) prompted us to look into its xylanolytic potential. While a number of xylanolytic *Paenibacillus* species have been isolated (8–10), most of the studies so far have been of structural-functional characterization of individual xylanolytic enzymes (11–14). However, a comprehensive approach investigating the complete xylan degradation potential of *Paenibacillus* species supported with in-depth genome analysis is not available thus far.

The present study is aimed at exploring the xylan degradation potential of the strain *Paenibacillus* sp. LS1. Here, we analyzed the growth and enzyme activity of the isolate using both cellulose and xylan substrates, which indicated that xylan is the preferred substrate. Further, whole-genome sequencing was performed to gain insights into the xylan-active enzyme repertoire and the associated metabolic pathway of the isolate. In addition, qRT-PCR analysis was performed to validate the involvement of the predicted carbohydrate-active enzymes (CAZymes) in xylan utilization. A comparative genome analysis employing cellulose and a xylan-active CAZyme repertoire of 238 distinct *Paenibacillus* species was also performed to understand the distribution and prevalence of the respective enzymes across the genus *Paenibacillus*. Furthermore, the orthologous average nucleotide identity (OrthoANIu) and *in silico* DNA-DNA hybridization (DDH) analysis were also performed to understand the novelty of strain LS1.

## RESULTS

### Cellulose and xylan degradation by *Paenibacillus* sp. LS1 through flask-based culture systems.

**FIG 1:**
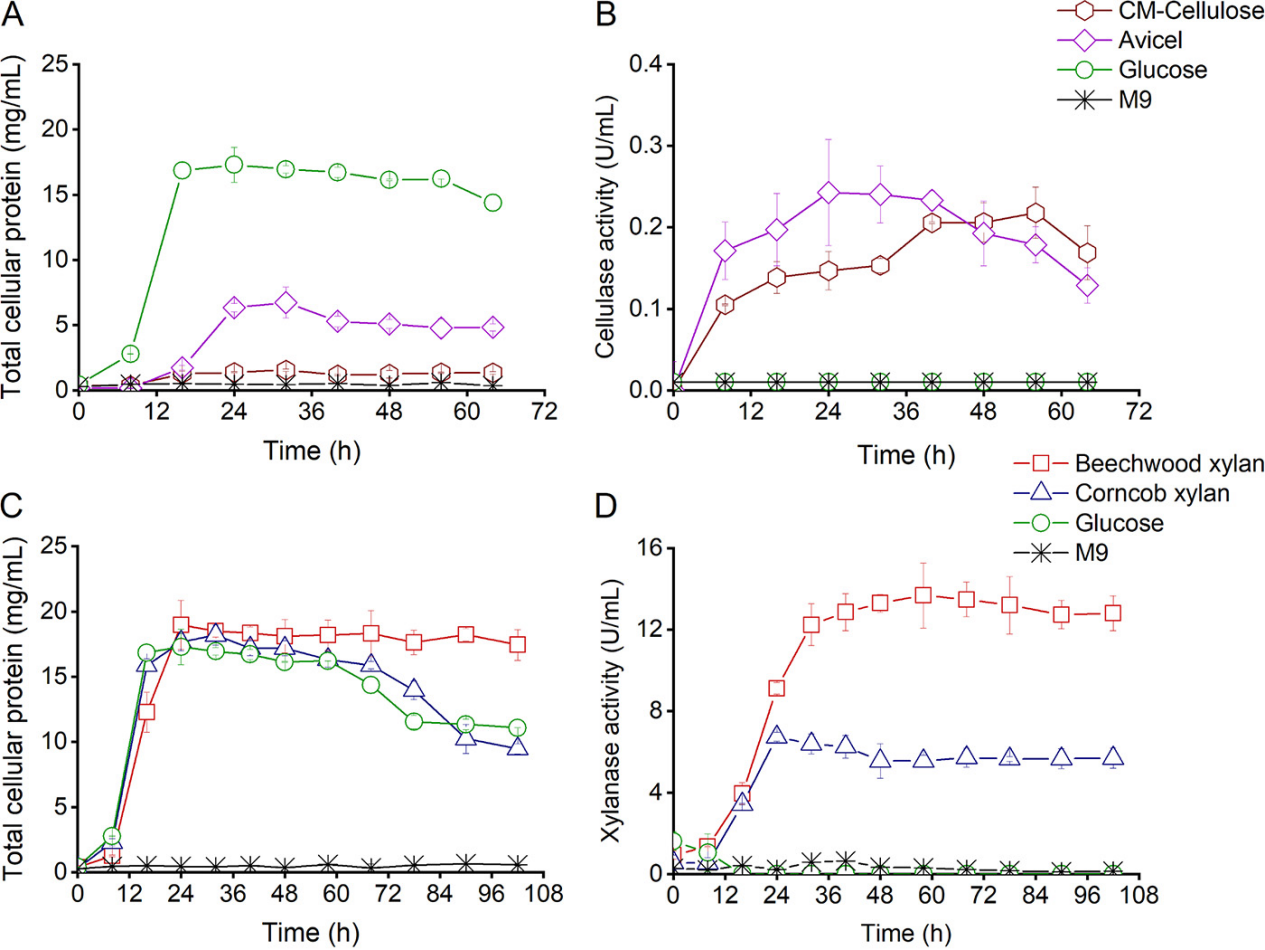
Cellulose and xylan degradation by *Paenibacillus* sp. LS1. (A) Growth of the isolate on different cellulose substrates measured in terms of total cellular protein. (B) Cellulase activity assessed for the same set of samples using cell-free culture supernatant collected over different time points. (C) Growth of the isolate on different xylan substrates measured in terms of total cellular protein. (D) Xylanase activity assessed for the same set of samples using cell-free culture supernatant collected over different time points. Experiments were performed in biological triplicates, and error bars represent standard deviations. Open hexagon, CM-cellulose; open diamond, Avicel; open square, beechwood xylan; open triangle, corncob xylan; open circle, glucose (positive control); star, M9 (negative control).

The growth of *Paenibacillus* sp. LS1 on M9 medium supplemented with different cellulose (Avicel and carboxymethyl [CM]-cellulose) and xylan substrates (beechwood and corncob xylan) was monitored. Samples were collected at regular time intervals to analyze the total cell protein (indication of growth) and cellulase/xylanase activity. The growth of *Paenibacillus* sp. LS1 on the substrates Avicel and CM-cellulose was compromised, as indicated by the total cellular protein content of 6.7 ± 1.2 mg/mL and 1.6 ± 0.1 mg/mL, respectively by 32 h (Fig. 1A). Also, strain LS1 displayed very low cellulase activity on the substrates Avicel and CM-cellulose (0.2 ± 0.06 U/mL) even after prolonged incubations (24 to 56 h) (Fig. 1B). However, the bacteria displayed identical growth patterns on both beechwood and corncob xylan, reaching the highest cellular protein content of up to 19 ± 1.8 mg/mL (by 24 h) and 18.2 ± 0.8 mg/mL (by 36 h), respectively (Fig. 1C). In agreement with the growth studies, *Paenibacillus* sp. LS1 exhibited predominantly higher xylanase activity than cellulase activity. While the isolate *Paenibacillus* sp. LS1 displayed maximum activity on xylan substrates, 2-fold higher activity was recorded on the substrate beechwood xylan (14 ± 1.6 U/mL) than corncob xylan (6.7 ± 0.2 U/mL) (Fig. 1D).

### General features of the *Paenibacillus* sp. LS1 genome.

The assembled genome of *Paenibacillus* sp. LS1 comprised a total of 40 contigs, with an *N*_50_ contig size of 0.43 Mb. The genome size was estimated to be 7.23 Mb, and the GC content was 45.7%. Two copies of 23S, a single copy each of the 16S and 5S rRNA genes, and 74 tRNA genes coding all 20 amino acids were identified. Genome annotation revealed a total of 6,642 protein-coding genes, 3,990 (60%) of which were functionally assigned. The function of the remaining 2,652 genes could not be predicted, and hence they were designated hypothetical. Rapid Annotations using Subsystems Technology (RAST) annotation classified these protein-coding genes into 26 categories of 342 subsystems (Fig. S2). The genome of *Paenibacillus* sp. LS1 included all 107 known housekeeping (marker) genes (15), therefore indicating the completeness of the genome (Table S2).

### The genome consists of a repertoire of xylan-active CAZymes.

**FIG 2:**
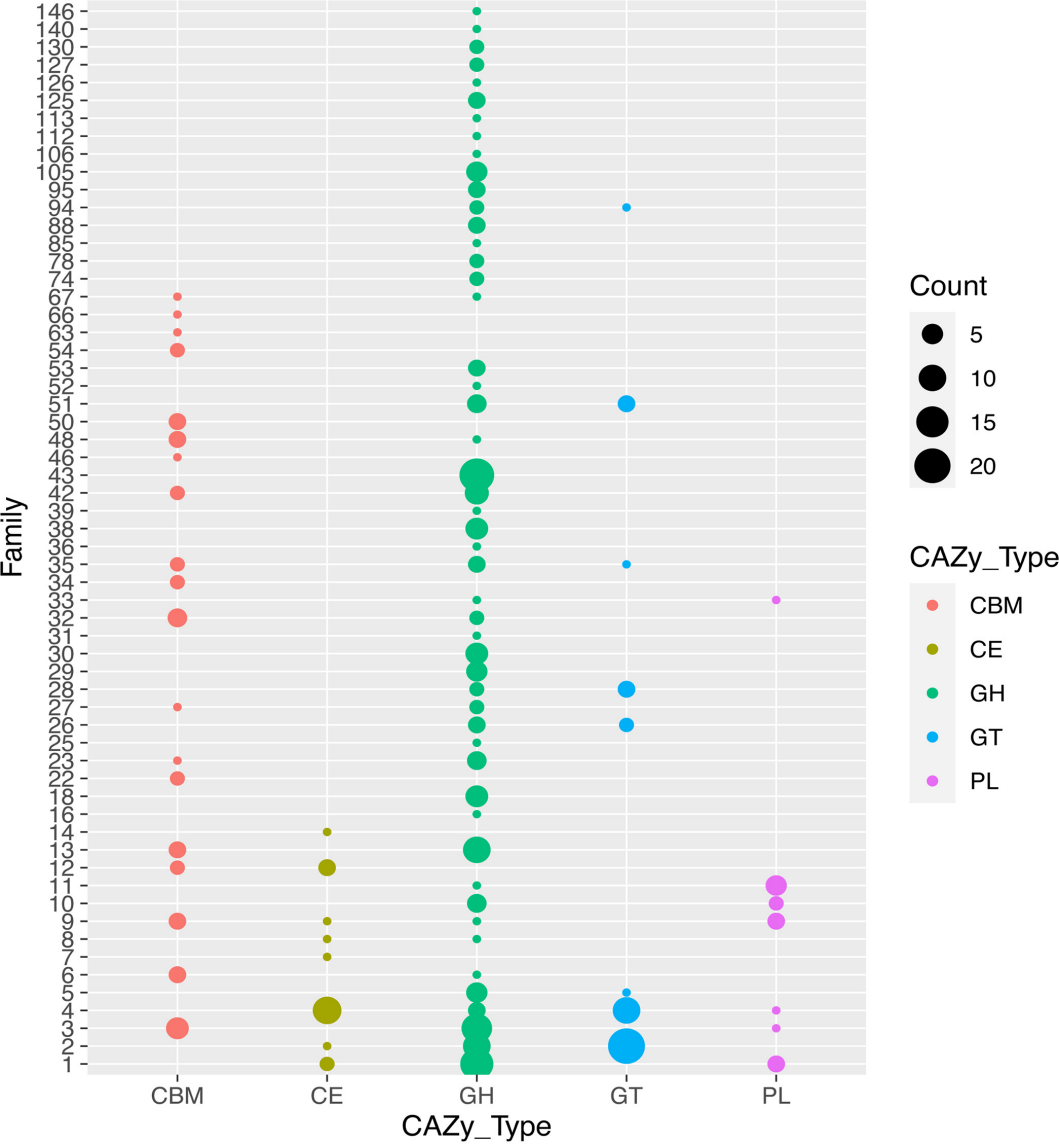
Family-wise distribution of different CAZyme classes in the genome of *Paenibacillus* sp. LS1. The different CAZyme classes shown are CBM, carbohydrate-binding modules; CE, carbohydrate esterases; GH, glycoside hydrolases; GT, glycosyl transferases; PL, polysaccharide lyases.

The CAZyme repertoire of *Paenibacillus* sp. LS1 consisted of 270 unique proteins consisting of 299 CAZyme domains. The genome of *Paenibacillus* sp. LS1 was dominated by 176 glycoside hydrolases (GH), followed by 43 carbohydrate-binding modules (CBM), 42 glycosyl transferases (GT), 21 carbohydrate esterases (CE), 16 polysaccharide lyases (PL) (Fig. 2), and 1 auxiliary activity (AA) family 7 protein. No putative AA10 lytic polysaccharide monooxygenases (LPMOs) were identified. The presence of representative CAZymes from families GH1, 2, 3, 5, 6, 8, 9, 10, 11, 30, 31, 39, 43, 48, 51, 67, and 74 among the glycoside hydrolases and CE1, 2, 3, 4, and 7 among the carbohydrate esterases suggests that the *Paenibacillus* sp. LS1 primarily targets lignocellulosic polysaccharides, in particular, hemicellulose (Fig. 2). Among the GHs, GH43 was the dominant family, with 18 representative members, which indicates the potential of the isolate to target arabinose moieties appended to xylans and pectins. Apart from this, the genome of *Paenibacillus* sp. LS1 also encoded cellulolytic CAZymes which consisted of four putative cellulases (GH5, PATRIC sequence ID peg.5091; GH6, peg.5652; GH9, peg.3676; GH48, peg.3677) and 10 β-1,4-glucosidases belonging to the GH1 and GH3 families (Fig. S3 and Table S4). In addition to this, genome analysis also confirmed the absence of CAZy families encoding lignin-degrading enzymes such as AA1 and AA2 in *Paenibacillus* sp. LS1. This was in line with the results obtained while screening the substrate preference, wherein the strain *Paenibacillus* sp. LS1 did not grow on the minimal medium supplemented with lignin (data not shown).

**FIG 3:**
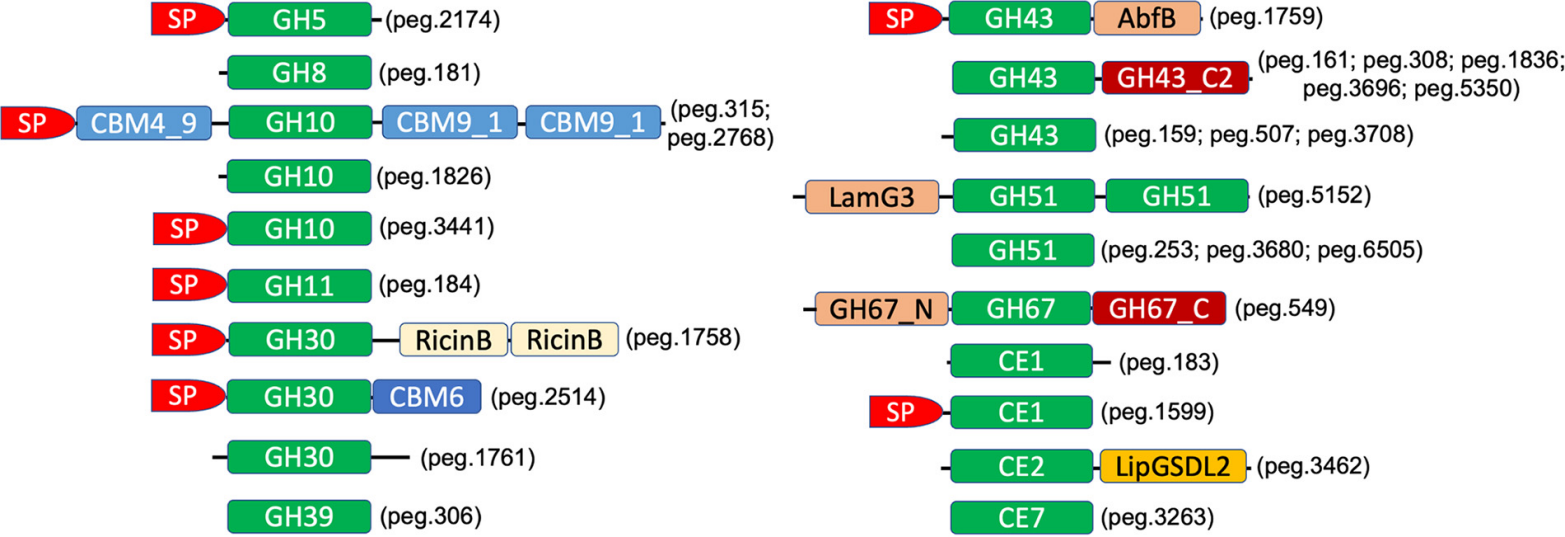
Schematic representation of the domain organization of the predicted xylan degrading/modifying CAZymes in *Paenibacillus* sp. LS1. The PATRIC sequence IDs (starting with “peg”) of the respective CAZymes with a particular domain architecture are indicated in parenthesis. SP, signal peptide; GH, glycoside hydrolase; CBM, carbohydrate-binding module; CE, carbohydrate esterase; SLH, S-layer homology domain; RicinB, ricin-type beta-trefoil lectin domain-like domain; AbfB, α-l-arabinofuranosidase B domain; GH43_C2, β-xylosidase C-terminal concanavalin A-like domain; LamG3, concanavalin A-like lectin/glucanase superfamily domain; GH67_N, glycoside hydrolase family 67 N-terminal domain; GH67_C, glycoside hydrolase family 67 C-terminal domain; LipGSDL2, GDSL-like lipase/acylhydrolase family.

**Xylan degradation.** The genome of *Paenibacillus* sp. LS1 comprises a complete arsenal of xylanolytic CAZymes capable of targeting different substitutions along with the xylose backbone of the polymer. The genome encodes 10 α-l-arabinofuranosidases, segregated into families GH30 (*n* = 2), GH43 (*n* = 4), and GH51 (*n* = 4), that can target and remove l-Araf moieties appended to the d-xylose residues. The GH30 α-l-arabinofuranosidases peg.1758 and peg.1761 shared very low sequence identity of 29.2% and 25.6% with the GH30 endoxylanase from Clostridium papyrosolvens C71 (PDB: 4FMV) (16) and the bifunctional GH30 xylanase B from Talaromyces cellulolyticus CF-2612 (17), respectively. The genome encoded 18 GH43 CAZymes, 4 of which were annotated as putative arabinoxylan arabinofuranohydrolases/α-l-arabinofuranosidases (peg.159, peg.507, peg.1759, and peg.3708). Three of the GH43 α-l-arabinofuranosidases in *Paenibacillus* sp. LS1 were single-domain proteins (Fig. 3); the exception was peg.1759, which had an N-terminal signal peptide and an α-l-arabinofuranosidase B (AbfB) domain at the C-terminal end along with the GH43 catalytic domain. Among the GH51 α-l-arabinofuranosidases, peg.5152 shared low sequence identity of 24% against the arabinofuranosidase from Thermobacillus xylanilyticus (PDB: 2VRQ) (18).

Removal of the d-MeGlcAp moieties can be mediated by the GH67 xylan α-1,2-glucuronosidase, peg.549. It is a tri-modular enzyme (Fig. 3) devoid of a signal peptide (Table S3), which indicates that the enzyme is not secreted outside the cell. It shared 63% identity with α-d-glucuronidase from Geobacillus stearothermophilus T-6 (PDB: 1MQP) (19).

Acetyl residues on the xylan backbone can be targeted by the CE2 (peg.3462) and CE7 (peg.3263) esterases encoded by *Paenibacillus* sp. LS1. The encoded CE2 (peg.3462) and CE7 (peg.3263) enzymes shared 46.2% and 54% identity against an acetyl xylan esterase (Est2A) from Butyrivibrio proteoclasticus (PDB: 3U37) (20) and acetyl xylan esterase 1 from *Thermoanaerobacterium* sp. strain JW/SL YS485 (PDB: 3FCY), respectively. Furthermore, the ferulate residues appended to the l-Araf moieties can be targeted by the two CE1 CAZymes, peg.183 and peg.1599, which shared 48.2% and 46.3% identity, respectively, against an endo-1,4-β-xylanase Z (a feruloyl esterase) from Acetivibrio thermocellus (PDB: 1JJF) (21). Among the esterases encoded, only peg.1599 comprised a signal peptide (Table S3).

The genome of *Paenibacillus* sp. LS1 encoded six endo-xylanases, segregated into GH5 (*n* = 1), GH10 (*n* = 4), and GH11 (*n* = 1), that can hydrolyze the internal β-1,4 glycosidic bonds in the xylan chains to generate xylooligosaccharides. Both GH5 and GH11 were unimodular proteins (Fig. 3), sharing 97.2% identity against a native family 5 xyloglucanase from Paenibacillus pabuli (PDB: 2JEP) (22) and 83.8% against a mesophilic xylanase A from Bacillus subtilis 1A1 (PDB: 1XXN) (23), respectively. Among the four GH10s, the unimodular xylanase peg.3441 shared 43.1% identity against a Caldicellulosiruptor danielii GH10 catalytic module (PDB ID: 6D5C) (24), and the tetramodular xylanase peg.315 shared 46.7% identity against a GH10 endo-β-1,4-xylanase (XynB) from Xanthomonas axonopodis pv. *citri* (PDB: 4PMX) (25). The genome further encoded a GH30 glucuronoarabinoxylan endo-1,4-β-xylanase, peg.2514, sharing 87.5% identity with Xyn30D from Paenibacillus barcinonensis (PDB: 4QAW) (26), known to be active on xylan chains highly substituted with methylglucuronic acid residues. All, except the GH10 xylanase peg.1826, consisted of an N-terminal signal peptide (Table S3), suggesting their involvement in extracellular xylan hydrolysis. In addition to the endo-xylanases, the genome of *Paenibacillus* sp. LS1 also comprised a GH8 oligosaccharide reducing-end xylanase (peg.181), which is reported to release xylose from xylooligosaccharides from the reducing end (14), thereby complementing the action of endo-xylanases.

The processing of xylooligosaccharides to monomeric xylose units could be mediated by the encoded β-1,4-xylosidases in *Paenibacillus* sp. LS1. The only GH39 β-1,4-xylosidase of *Paenibacillus* sp. LS1, peg.306, shared 35.7% identity with GH39 β-xylosidase XynB1 from Geobacillus stearothermophilus (PDB: 2BS9) (27) and lacked signal peptide (Table S3). Overall, five GH43 β-1,4-xylosidases from *Paenibacillus* sp. LS1 shared a common domain architecture, comprising an N-terminal catalytic domain and a C-terminal noncatalytic concanavalin-A-like domain (Fig. 3 and Table S3). However, the majority of the GH43 enzymes are devoid of signal peptide (Table S3), suggesting that the conversion of xylooligosaccharides to xylose happens inside the cell.

### Transport and metabolism of xylan degradation products in *Paenibacillus* sp. LS1.

**FIG 4:**
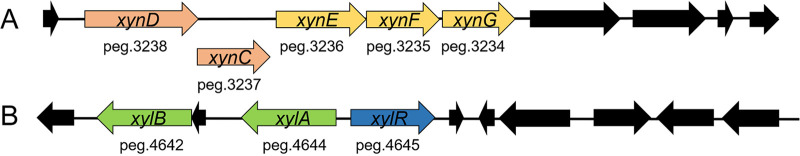
(A and B) Genetic loci in *Paenibacillus* sp. LS1 showing the position of genes involved in the *xynEFG* ABC transport system (A) and genes involved in xylooligosaccharide metabolism and regulation (B). Yellow arrows represent the different genes that constitute the *xynEFG* ABC transporter. Orange arrows represent the *xynDC* two-component system, which senses and regulates xylooligosaccharide uptake. The blue arrow represents the *xylR* gene, which regulates the uptake and metabolism of xylooligosaccharides. Green arrows represent the genes involved in xylose metabolism, and black arrows represent unrelated genes. The PATRIC sequence IDs of the individual genes are indicated beneath the arrows.

Homologs for the xylooligosaccharide ABC transporter XynEFG from Geobacillus stearothermophilus T-6 (28) were identified in *Paenibacillus* sp. LS1. The proteins peg.3236, peg.3235, and peg.3234 shared 58%, 63%, and 67% sequence identity with the sugar-binding protein XynE (ABI49932.1) and the permeases XynF (ABI49933.1) and XynG (ABI49934.1) of the XynEFG transporter, respectively. These genes were identified as part of the *xynDCEFG* operon (Fig. 4A), where *xynD* and *xynE* were the two-component system, located upstream of the transporter genes. The homologs peg.3238 and peg.3237 shared 40% and 50% sequence identity, respectively, with XynD and XynC of *G. stearothermophilus* T-6. Furthermore, the presence of xylose isomerase (XylA; peg.4644) and xylulose kinase (XylB; peg.4642) confirms the ability of *Paenibacillus* sp. LS1 to metabolize xylose via the xylose isomerase pathway (29). The homologs were confirmed by the presence of the transcriptional factor, XylR (ROK family protein), peg.4645 in the same genetic loci, as reported previously (30) (Fig. 4B).

### Expression profiling of xylan-active genes during growth of *Paenibacillus* sp. LS1 on different xylan substrates.

**FIG 5:**
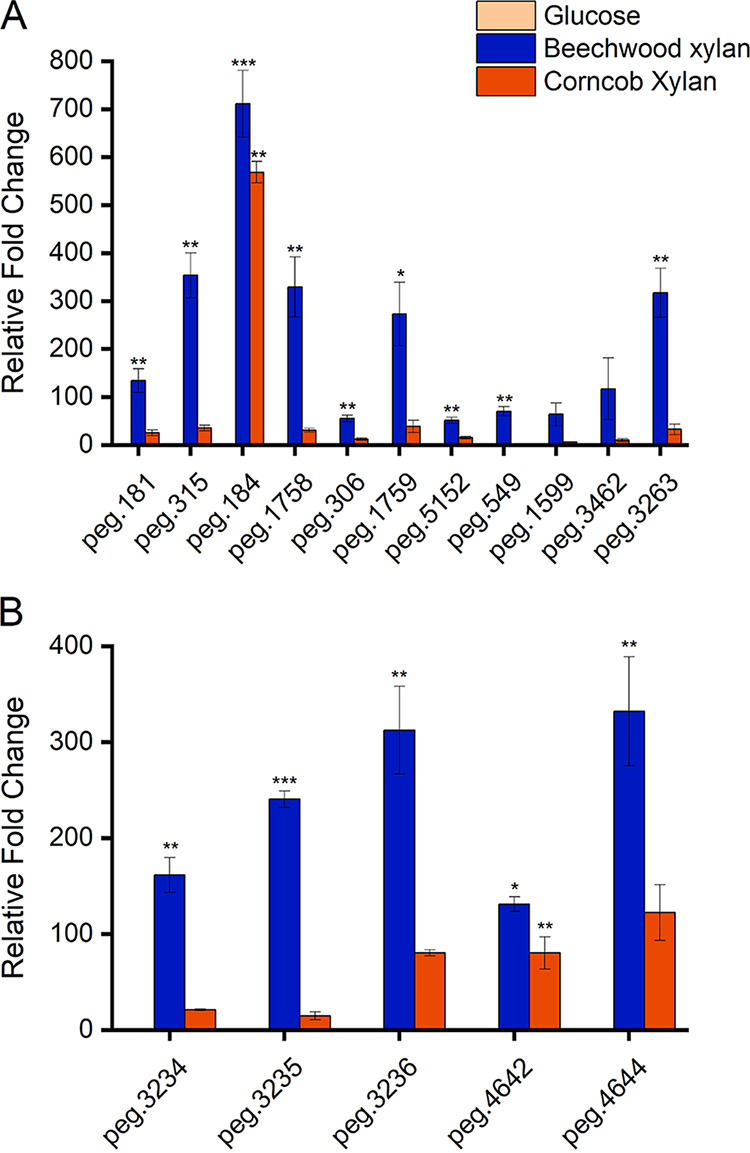
(A and B) Comparison of expression levels of genes involved in xylan degradation/modification (A) and xylooligosaccharide transport and metabolism (B) using qRT-PCR. Gene expression was analyzed in the presence of beechwood xylan and corncob xylan as the sole carbon source, while glucose was used as the control. Gene expression levels are shown in terms of relative fold change. Bars indicate the mean ± standard deviation fold change, and the asterisks indicate *P* values, as follows: ***, *P < *0.001; **, *P < *0.01; *, *P < *0.05. All experiments were performed in biological triplicates.

qRT-PCR analysis confirmed the expression of selected genes while growth of the strain LS1 on the xylan substrates compared to the control (glucose). Notably, the gene expression was substantially higher in the presence of the substrate beechwood xylan than corncob xylan (Fig. 5). Most of the genes, including the GH10 xylanases (peg.181 and peg.315), GH39 β-1,4-xylosidase (peg.306), α-l-arabinofuranosidases from the GH30 (peg.1758) and GH43 (peg.1759) families, and xylan esterases (peg.1599, peg.3462, and peg.3263) showed a ≥5-fold increase in the gene expression when grown on beechwood xylan compared to corncob xylan. The α-l-arabinofuranosidase from the GH51 family (peg.5152) displayed a 3-fold increase in gene expression when grown on beechwood xylan compared to corncob xylan, while the gene expression of the only GH11 xylanase (peg.184) was 1.3-fold higher in beechwood xylan than corncob xylan. Notably, expression of the enzyme GH67 xylan α-1,2-glucuronosidase (peg.549) was observed only in beechwood xylan. Similar to the xylan-active CAZymes, the xylooligosaccharide transporter components and the respective metabolic genes also showed higher expression in the presence of beechwood xylan. The transporter component genes, peg.3234, peg.3235, and peg.3236 showed 8, 16, and 4 times increases in the relative fold change of expression in the presence of beechwood xylan, while the metabolic genes, xylose isomerase (peg.4644), and xylulose kinase (peg.4642) showed 2 and 3 times higher expression.

### The *Paenibacillus* sp. LS1 secretomes displayed higher specificity to beechwood xylan.

**FIG 6:**
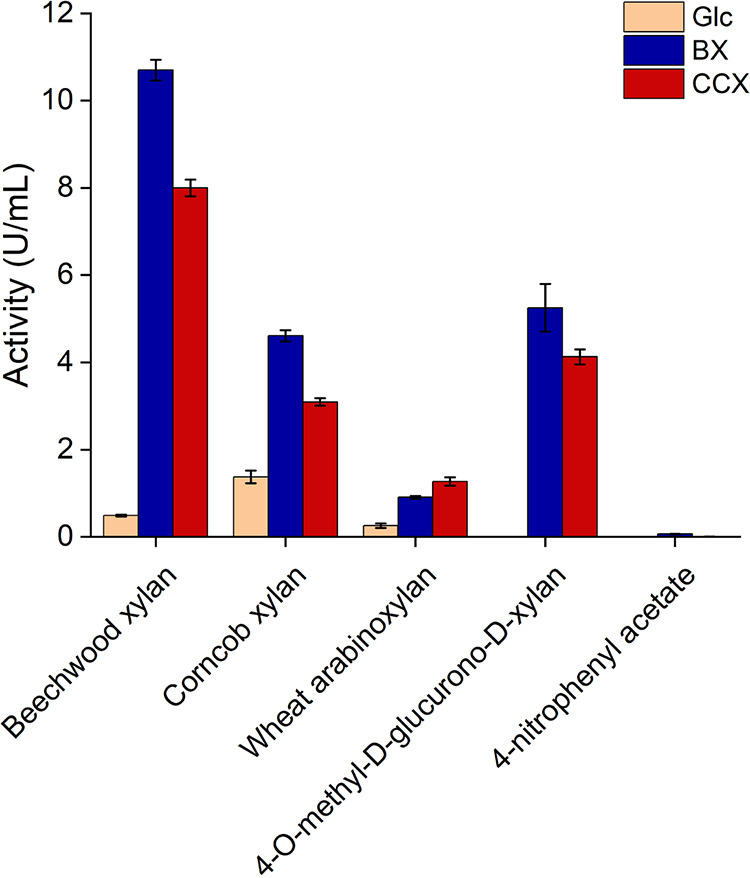
Substrate specificity of xylan-active secretomes on different xylan substrates. Glc, BX, and CCX represent the secretomes collected over 0.5% glucose, beechwood xylan, and corncob xylan, respectively. Experiments were performed in biological triplicates, and error bars represent standard deviations.

The *Paenibacillus* sp. LS1 secretomes collected over beechwood and corncob xylan were tested on different xylan substrates to understand the substrate specificity. Both the secretomes displayed the highest overall xylanase activity of 10.7 U/mL and 8 U/mL, respectively, on the beechwood xylan substrate (Fig. 6). Of note, the secretome collected over beechwood substrate showed the highest xylanase activity on corncob xylan. This indicates that the secretome collected over the beechwood xylan substrate is rich in enzymes that could target both hardwood xylan and xylan from the graminaceous plants. The α-glucuronidase activity against 4-*O*-methyl-d-glucurono-d-xylan was slightly higher for the secretome obtained from beechwood xylan (5.3 U/mL) than corncob xylan (4.1 U/mL). Similarly, the arabinoxylanase activity tested over wheat arabinoxylan was slightly higher for the secretome obtained from corncob xylan substrate (1.2 U/mL) than beechwood xylan (0.9 U/mL) (Fig. 6). Notably, feeble activity on 4-nitrophenyl acetate was observed for the secretome collected over beechwood xylan (0.07 U/mL), suggesting weak acetyl xylan esterase activity, while it was negligible for the secretome collected over corncob xylan substrate (Fig. 6).

### Comparative genome analysis.

Genome-based relatedness was estimated in terms of OrthoANIu and *in silico* DDH. Among the phylogenetic neighbors, genomes for the *Paenibacillus* strains P. tundrae A10b^T^, P. tylopili MK2^T^, P. cucumis AP-115^T^, and P. oceanisediminis L10^T^ were not available in the NCBI genome database, and hence these were not considered for calculating OrthoANIu and DDH values. On the other hand, in a comparison against the closest phylogenetic neighbors, P. amylolyticus NBRC 15957^T^ and P. xylanexedens DSM 21292^T^, OrthoANIu values of 92.17% and 92.28% and DDH values of 46.9% and 47.1%, respectively were obtained (Table S5). Furthermore, the OrthoANIu and DDH values obtained for the other phylogenetic neighbors, P. polysaccharolyticus BL9^T^, *P. pabuli* NBRC 13638^T^, P. xylanilyticus LMG 21957^T^, and P. taichungensis DSM 19942^T^ were in the range of 78 to 82% and 22 to 25.5%, respectively (Table S5).

**FIG 7:**
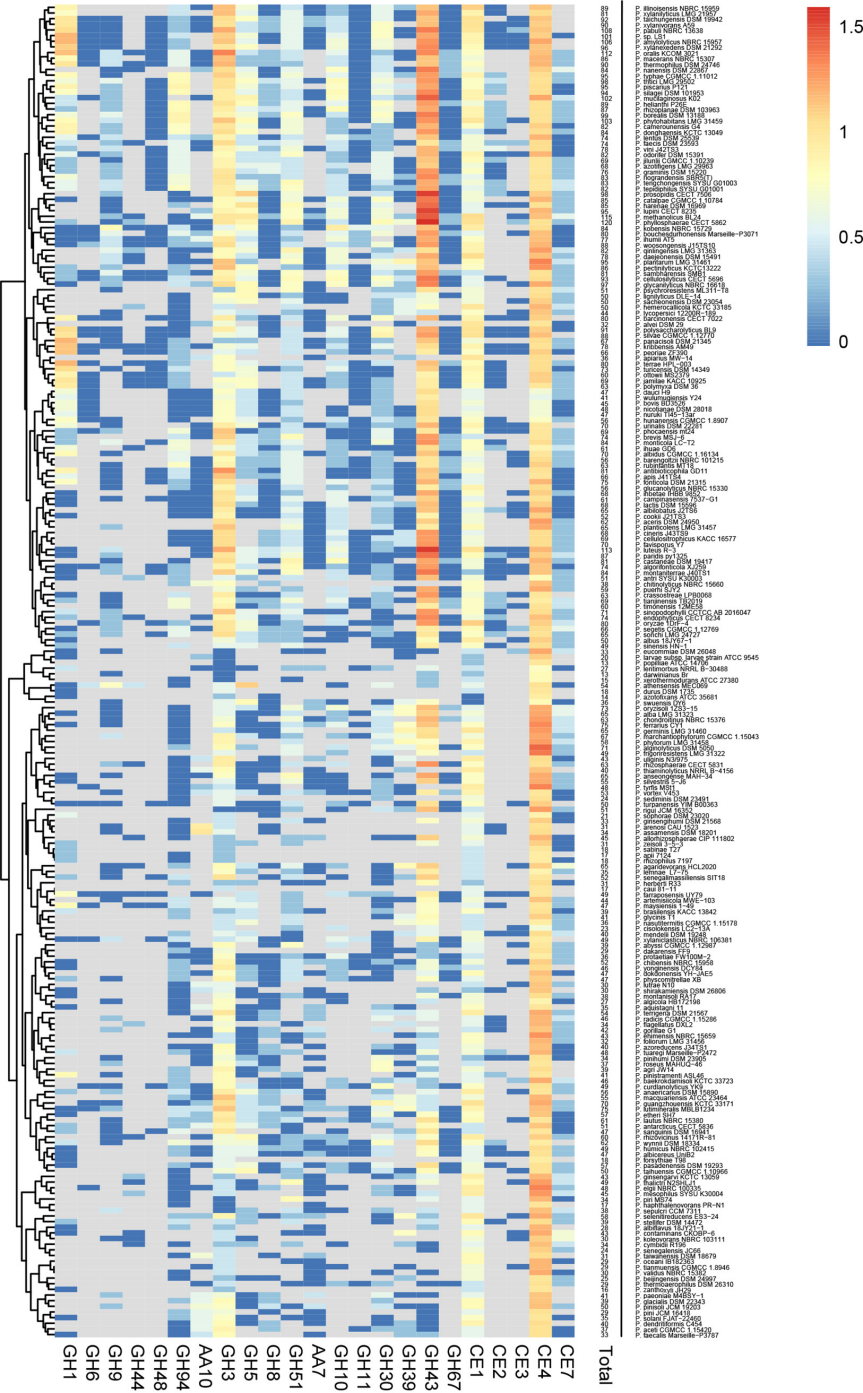
Functionally clustered heat map based on the predicted cellulose and xylan-active CAZymes of the 238 *Paenibacillus* species. The key displays the log-transformed counts of the CAZymes.

Comparative genome analysis of 238 distinct *Paenibacillus* species was performed to understand the distribution of cellulose- and xylan-active CAZymes across the genus (Fig. 7). A clustered heat map was generated based on the predicted cellulose/xylan-active CAZymes of the *Paenibacillus* species. Out of 238 genomes, 91 genomes lack cellulases belonging to the families GH6, GH9, GH44, and GH48. The genome of *Paenibacillus* sp. LS1 encoded only 3 cellulases each from the families GH6, GH9, and GH48. A similar occurrence was also observed for 39 other *Paenibacillus* genomes as well. Only 28 genomes encoded ≥4 cellulases, with the highest being encoded by P. athensensis MEC069 (12 cellulases), P. kobensis NBRC 15729 (10 cellulases), P. sambharensis SMB1, and P. woosongensis J15TS10 (7 cellulases). In addition to this, 106 *Paenibacillus* genomes lacked β-1,4-glucosidases (GH1), and 56 of these genomes also did not encode cellulases, making them strictly noncellulolytic representatives of the *Paenibacillus* genus.

Only 41 out of the 238 *Paenibacillus* genomes lacked xylanases, i.e., representatives from GH10, GH11, and GH30. In total, 19 species out of 238 were devoid of β-1,4-xylosidases (representatives from GH39 and GH43), while 18 species completely lacked xylanases as well as β-1,4-xylosidases, making them the strict nonxylanolytic representatives among the *Paenibacillus* species. It is intriguing to note that among the CAZyme classes that target specific substitution moieties in xylan, the enzymes xylan α-1,2-glucuronosidase (GH67) and α-l-arabinofuranosidase (GH30 and GH43) were present in 131 and 212 *Paenibacillus* genomes, respectively. This showed the prevalence of α-l-arabinofuranosidases (targets arabinose moieties in xylan—abundant in graminaceous plants) over α-1,2-glucuronosidases (targets methyl-glucuronic acid residues—abundant in hardwood species). While true acetyl-xylan esterases (CE2 and CE3) were limited to only 108 of the total *Paenibacillus* genomes under study, the multisubstrate-specific classes such as CE1 and CE4 were abundant in all *Paenibacillus* species, and CE7 was present in 191 genomes.

Among the CAZyme families with mixed specificity toward cellulose and/or xylan i.e., GH3, GH5, GH8, GH51, and AA7, GH3 was the most abundant CAZyme family, which was absent only in the genome of P. farraposensis UY79, out of the 238 *Paenibacillus* genomes. However, in the case of auxiliary activity family of proteins, it was observed that 117 *Paenibacillus* genomes encoded the AA7 class of enzymes, which has been previously reported to be from fungal origin and to be active on cello- and xylo-oligosaccharides (31, 32). Of note, most of the *Paenibacillus* genomes (135 genomes) lack the AA10 family of LPMOs, and only 17 genomes encoded ≥4 AA10 LPMOs.

## DISCUSSION

*Paenibacillus* species are ubiquitous bacteria, thriving over disparate environments (33). They are known to produce hydrolytic enzymes for the deconstruction of eukaryotic cell walls and are major degraders of plant cell wall polysaccharides, particularly cellulose and hemicellulose (34). However, a genome-level comparison conducted to understand the overall enzyme complement involved in cellulose and hemicellulose (xylan in particular) utilization by the genus *Paenibacillus* is essential in view of the growing demand for biobased fuels. In this study, we attempted to address these gaps using biochemical, molecular, and genomic approaches. The organism under study, *Paenibacillus* sp. LS1, is a novel species, supported by the ANI and *in silico* DDH analysis performed against its phylogenetic neighbors. The genome relatedness values were below the assigned cutoffs for ANI (≤95%) and DDH (≤70%), therefore supporting our claim of *Paenibacillus* sp. LS1 being a novel species in the *Paenibacillus* genus.

### Xylan degradation is a predominant feature across the genus *Paenibacillus*.

To date, there are 341 distinct species in the genus *Paenibacillus* that have been taxonomically characterized and validly published (LPSN, https://www.bacterio.net/). Among these, the genome sequence was available for 237 distinct species (Table S6), and these were considered for comparative genome analysis with a focus on understanding the prevalence and distribution of cellulose/xylan-active CAZymes.

Comparative genome analysis based on the CAZyme profiling clearly demarcated a compromise on cellulose degradation potential across the genus *Paenibacillus*. This is evident from the fact that 91 *Paenibacillus* spp. do not encode any cellulases, while 56 species completely lack any cellulose-active CAZymes in their genomes. Only 28 out of 238 genomes harbored ≥4 cellulases. In contrast, 136 and 181 *Paenibacillus* genomes encoded ≥4 xylanases and β-1,4-xylosidases, strongly suggesting their preference for the xylan substrates. In line with this, the isolate *Paenibacillus* sp. LS1 displayed maximum activity on xylan substrates (14 ± 1.6 U/mL was the highest over beechwood xylan). Of note, the activity displayed by *Paenibacillus* sp. LS1 was higher than the activity achieved over the same substrate (3.66 U/mL at 72 h) by P. xylanivorans A59 (10, 35). In addition, in the current study it was noticed that a majority of *Paenibacillus* genomes do not encode LPMOs (only 103 out of 238 *Paenibacillus* genomes encode AA10 LPMO). In general, LPMOs are known for boosting the activity of GHs (3). Several families of these proteins have been described, with different substrate specificities and origins (36). Although the xylan-active LPMOs were initially reported to be produced from the fungus Pycnoporus coccineus (37), later studies indicated the occurrence of these families of LPMOs in the actinobacterium Kitasatospora papulosa (38). Interestingly, the comparative genome analysis also revealed 31 *Paenibacillus* spp. that possessed at least one xylanase and one AA10 LPMO but completely lacked cellulases in their genomes. Such a unique CAZyme repertoire may indicate the possible implications that these encoded AA10 LPMOs might have on xylan degradation by these 31 *Paenibacillus* spp. and requires further investigation. Considering the fact that a majority of *Paenibacillus* spp. have a preference for xylan substrates, it would be interesting to validate the substrate preference of LPMOs from the strict xylan-utilizing *Paenibacillus* isolates, which might help expand the xylan-active LPMO repertoire.

*Paenibacillus* sp. LS1 is unique in having at least one representative member from each xylan-active CAZyme family. This unique feature was shared by only five other *Paenibacillus* species, namely, P. faecis DSM 23593, P. montaniterrae J40TS1, P. paridis py1325, P. phocaensis mt24, and P. phytohabitans LMG 31459. This confirms the genomic potential of these bacteria for complete deconstruction of xylan. However, in the hierarchical clustering, *Paenibacillus* sp. LS1 did not share the same clade with either of these species (Fig. 7). Instead, *Paenibacillus* sp. LS1 was clustered together with its nearest phylogenetic neighbors within the same clade. The reason for this could be sharing a similar CAZyme profile with its nearest phylogenetic neighbors, *P. amylolyticus* NBRC 15957 and *P. xylanexedens* DSM 21292. The results from the comparative genome analysis collectively confirm that the members of the *Paenibacillus* genus are predominantly xylan degraders, irrespective of the habitat in which they thrive.

### *Paenibacillus* sp. LS1 prefers complex hardwood xylan.

Hardwood xylan (predominantly seen in dicots) such as beechwood xylan constitutes 4-*O*-methyl glucuronyl residues and acetyl esters conjugated with the xylose residues. Such an arrangement of the polymeric chain makes hardwood xylan more complex than that of monocot/grass xylan such as corncob xylan, wherein the l-arabinofuranosyl moieties are prevalent (some of which are modified to ferulate esters) (39). The genome of *Paenibacillus* sp. LS1 encodes a complete CAZyme arsenal for efficient deconstruction of xylan (Fig. 3). Interestingly, the results from both growth and activity studies, along with qRT-PCR-based expression analysis of selected xylan-active genes, infer a strong preference for beechwood xylan over the corncob xylan. The presence of 4-*O*-methyl glucuronyl residues and acetyl esters in hardwood xylan (such as beechwood xylan) justifies the high expression of the GH67 xylan α-1,2-glucuronosidase gene (peg.549) and the acetyl xylan esterase genes (CE2, peg.3462; CE7, peg.3463) on the substrate. Interestingly, peg.1599, a CE1 family CAZyme, also showed higher expression on beechwood xylan than on corncob xylan, and BLAST analysis identified peg.1599 as a putative feruloyl esterase (targets ferulic acid esters linked to arabinoxylans in monocots). However, it is notable that the CE1 family consists of enzymes with multiple activities such as acetyl xylan esterase and feruloyl esterase, and therefore, the actual role of peg.1599 needs further investigation. Also, it should be noted that the gene expression levels of the encoded acetyl xylan esterases (Fig. 5 and Table S3) and their activity (at least from the complex secretomes in this study) (Fig. 6) could not be correlated. This could be due to the absence of signal peptide in the majority of the acetyl xylan esterases produced by the strain *Paenibacillus* sp. LS1 (Fig. 3 and Table S3), suggesting that they are not secreted extracellularly and hence explaining the feeble activity.

Even the predicted arabinofuranosidases, peg.1758 (GH30), peg.1759 (GH43), and peg.5152 (GH51), displayed high expression in the presence of beechwood xylan, while this may seem unlikely since these CAZymes generally act upon arabinoxylans in monocots. However, GH30, GH43, and GH51 are also multisubstrate-specific families that may exhibit endo-β-1,4-xylanase (GH30 and GH51) and β-1,4-xylosidase (GH43 and GH51) activities. Therefore, the observed high expression for peg.1758, peg.1759, and peg.5152 may demarcate more distinct activities than predicted. This is supported by the fact that the secretomes displayed relatively low arabinoxylanase or arabinofuranosidase activity on the wheat arabinoxylan (Fig. 6). Overall, only two of the predicted arabinofuranosidases (the GH30 enzyme, peg.1758, and the GH43 enzyme, peg.1759) had an N-terminal signal peptide (Fig. 3 and Table S3), suggesting their possible presence in the secretome. However, considering the multisubstrate specificity of GH30 and GH43 enzymes, it could be inferred that the secreted enzymes might have low arabinofuranosidase activity. Furthermore, the GH10 xylanases, peg.181 and peg.315, and the GH39 β-1,4-xylosidase, peg.306, also showed a preference toward beechwood xylan, displaying around 5 to 10 times higher expression in beechwood xylan than corncob xylan. The higher gene expression could be due to the presence of specific substitutions in the hardwood xylan. In line with this, expression of the gene peg.549 (α-1,2-glucuronosidase) can be observed only in the presence of beechwood xylan (Fig. 5).

In a different experiment, we tried to probe the α-glucuronosidase activity for the secretomes collected over both beechwood and corncob xylan substrates. Interestingly, the secretome collected over the corncob xylan also displayed a significant α-glucuronosidase activity (Fig. 6), suggesting the possible occurrence of enzymes with similar activity other than peg. 549, which was expressed only in the presence of beechwood xylan. Therefore, it would be interesting to identify the candidate enzyme(s) responsible for the α-glucuronosidase activity of the secretome collected over corncob xylan. The only gene to show relatively similar expression on both substrates was the GH11 xylanase (peg.184). The results collectively indicate that strain LS1 prefers complex substrates, such as beechwood xylan.

### *Paenibacillus* sp. LS1 encodes the machinery to transport and metabolize xylooligosaccharides.

The transport of xylose and xylooligosaccharides in *Firmicutes* has been previously reported for bacteria belonging to the *Bacillaceae* and *Clostridiaceae* families (30). Also, transcriptomic analysis of *Paenibacillus* sp. JDR-2 identified several transport system components that were upregulated during growth over different xylan substrates (39). The genome analysis revealed the absence of the d-xylose transport system in *Paenibacillus* sp. LS1. However, homologues of the xylooligosaccharide ABC transporter XynEFG, which was previously reported for Geobacillus stearothermophilus T-6 (29), were identified in *Paenibacillus* sp. LS1. Further, the genes encoding the transporter components were identified as part of the *xynDCEFG* operon (Fig. 4A), where *xynD* and *xynC* were the two-component system, acting as a sensor histidine kinase (senses the presence of xylooligosaccharides in the external environment through signal transduction) and a response regulator (controlling the expression of the transporter genes), respectively (29). The presence of the complete operon confirms the plausible functional role of this transport system in the uptake of xylooligosaccharides by *Paenibacillus* sp. LS1.

**FIG 8:**
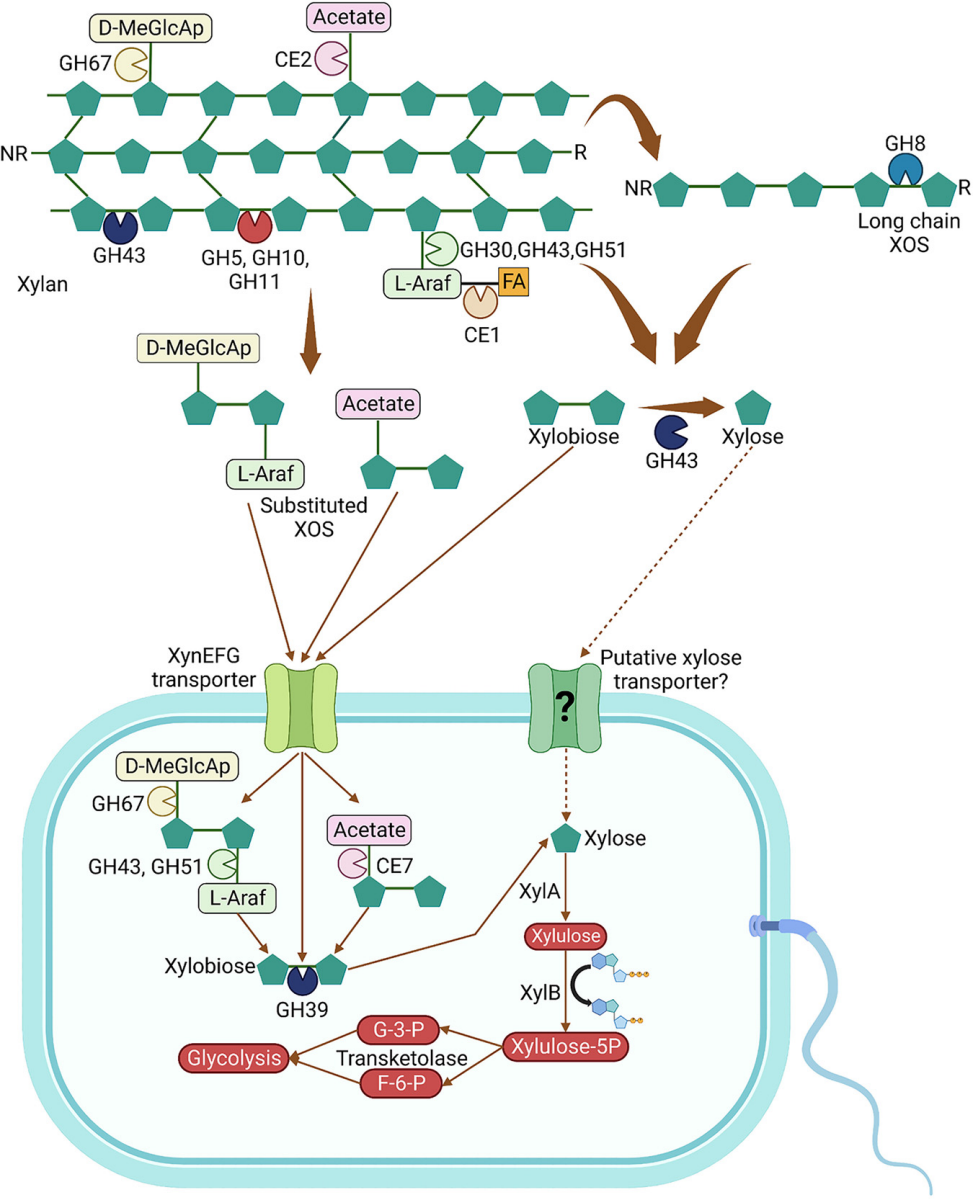
Proposed pathway for xylan degradation and metabolism in *Paenibacillus* sp. LS1. Solid linkers/arrows represent confirmed pathways, while the dotted lines infer that the pathway is hypothetical. The CAZymes involved in xylan degradation/modification are represented as cartoons with different colors as per their activities: β-1,4-endoxylanases (red), reducing end oligo-xylanase (light blue), β-1,4-xylosidases (dark blue), α-1,2-glucuronosidase (yellow), acetyl xylan esterase (pink), α-l-arabinofuranosidases (light green), and feruloyl esterase (orange). NR, nonreducing end; R, reducing end; GH, glycoside hydrolase; CE, carbohydrate esterase; d-MeGlcAp, 4-*O*-methyl glucuronyl residues; l-Araf, l-arabinofuranosyl; FA, ferulic acid; XOS, xylooligosaccharides; XylA, xylose isomerase; XylB, xylulose kinase; G-3-P, glyceraldehyde 3-phosphate; F-6-P, fructose 6-phosphate. This figure was prepared using Biorender (app.biorender.com).

Furthermore, *Paenibacillus* sp. LS1 employs the xylose isomerase pathway (29) to metabolize the imported xylooligosaccharides, which can be further degraded to xylose by cytoplasmic xylosidases. The identified xylose isomerase (XylA; peg.4644) of *Paenibacillus* sp. LS1 can mediate conversion of xylose to xylulose, which can then be phosphorylated to xylulose 5-phosphate by xylulose kinase (XylB; peg.4642). Furthermore, the presence of the transcriptional factor, XylR (ROK family protein), peg.4645 in the same genetic loci as XylA and XylB, confirms the operon as functional, as reported previously (30) (Fig. 4B). In addition to this, the xylulose 5-phosphate can be acted upon by transketolase (peg.3941) to generate glyceraldehyde 3-phosphate and fructose 6-phosphate, which can ultimately enter the Embden-Meyerhof-Parnas pathway (Fig. 8).

The genes encoding the transporter components and metabolic enzymes were analyzed for expression using qRT-PCR. Similar to the xylan-active CAZymes, the transporter components and the metabolic genes also displayed higher fold change in the presence of beechwood xylan than corncob xylan. Transporter components are specific to the molecule that they are binding to and ultimately transporting inside the cell. Significantly high upregulation of the transporter components (peg.3234, peg.3235, and peg.3236) indicates greater preference for the product(s) generated from the hydrolysis of beechwood xylan than corncob xylan. It would be interesting to undertake a real-time analysis of the transporter components in the presence of different xylooligosaccharide substrates, both linear and decorated, in order to understand the finer details of the substrate promiscuity of the transporter system. Additionally, the higher expression of the metabolic genes is indicative of the internalized product being efficiently hydrolyzed to xylose by the cytoplasmic enzymes and, therefore, upregulation of the metabolic enzymes. Collectively, genomic insights supported by expression analysis by qRT-PCR confirm the involvement of the predicted ABC transport system and metabolic enzymes in the transport and metabolism of xylooligosaccharides by the isolate *Paenibacillus* sp. LS1.

**Conclusion.** *Paenibacillus* sp. LS1 is a novel bacterium capable of efficient xylan degradation with the assistance of a complete arsenal of xylan-active CAZymes encoded in its genome. However, the strain was inefficient at utilizing different cellulosic substrates, evident from the growth and degradation studies and also supported by genome analysis. Growth and degradation studies of xylan, along with qRT-PCR analysis of the selected genes, affirms the preference for complex xylan substrates such as hardwood xylan by strain LS1. It also encodes a functional transport system for the internalization of xylooligosaccharides into the cell. In addition to this, comparative genome analysis revealed the prevalence of xylan-active CAZymes across the genus *Paenibacillus*. These results taken together indicate the possible application of the xylanolytic enzymes from *Paenibacillus* sp. LS1 or the other *Paenibacillus* species in lignocellulosic biorefineries, not only as pretreatment agents but also for generation of the value-added products.

## MATERIALS AND METHODS

### Bacterial strain, chemicals, reagents, and kits.

*Paenibacillus* sp. LS1 was isolated from a decaying wood sample collected from Lachung village, North Sikkim district (27°40′13″N, 88°43′37″E), Sikkim, India (7). Beechwood and corncob xylan were procured from Sisco Research Laboratories Pvt. Ltd., Mumbai, India. Avicel, CM-cellulose, 4-*O*-methyl-d-glucurono-d-xylan, and 4-nitrophenyl acetate were procured from Sigma-Aldrich, USA. All the chemicals required for minimal medium, Luria-Bertani broth, and 3,5-dinitrosalicylic acid (DNS) reagent preparation were procured from HiMedia Laboratories, Mumbai, India, unless otherwise specified. Bradford reagent and TRI reagent were procured from Sigma-Aldrich, USA. The Qubit double-stranded DNA (dsDNA) high-sensitivity (HS) assay kit was procured from Thermo Fisher Scientific, USA.

### Utilization of different cellulose and xylan substrates.

Growth studies of *Paenibacillus* sp. LS1 were performed in M9 minimal medium supplemented with different cellulose (0.5% each of Avicel and CM-cellulose) and xylan substrates (0.5% each of beechwood and corncob xylan). While, M9 medium and M9 supplemented with 0.5% glucose were used as negative and positive controls, respectively. The primary inoculum was prepared by growing the isolate in 10 mL Luria Bertani (LB) broth for 24 h at 28°C. The culture was harvested, and the cell pellet was washed thoroughly with M9 medium. Further, the pellet was resuspended in 10 mL M9 medium, from which 0.5% of the culture was inoculated into the different experimental flasks. All experiments were performed in biological triplicates. Culture (1 mL) from each flask was collected at different time intervals and centrifuged at 10,000 rpm for 15 min. Pellet and supernatant were collected separately and stored at −20°C until further analysis.

**(i) Estimation of total cell protein.** Growth of the isolate was analyzed by estimating the total cell protein in the culture pellet collected at different time points as described previously (7). The pellets were treated with 0.2 N NaOH and boiled at 120°C for 10 min for cell lysis. This was followed by centrifugation at 12,000 rpm for 15 min at 4°C. Total cell protein was estimated using the standard Bradford method, essentially as described by the manufacturer’s protocol.

**(ii) Estimation of extracellular cellulase and xylanase activity.** Reducing end assay to estimate cellulase and xylanase activity was performed using the DNS method (40) with slight modifications. A 200-μL reaction mixture consisting of 50 mM sodium phosphate (pH 7.0), 0.5% beechwood xylan or 0.5% CM-cellulose, and 50 μL of the enzyme cocktail was incubated at 37°C for 1 h with shaking at 800 rpm. The samples were centrifuged at 10,000 rpm for 15 min at 4°C. Then 40 μL of the clear supernatant was analyzed by adding 300 μL of the DNS reagent (1% 3,5-dinitrosalicylic acid, 2% NaOH, and 30% Na-K tartrate) and was boiled at 100°C for 15 min. The samples were cooled to room temperature for 5 min, and the absorbance was measured at 540 nm. The amount of reducing sugar generated was calculated using a glucose or xylose standard curve. One unit was defined as the amount of enzyme that liberated 1 μmol of reducing sugar per min.

### Genome sequencing, assembly, and annotation.

*Paenibacillus* sp. LS1 was grown in LB broth for 24 h, and the harvested culture pellet was used for DNA isolation using the standard procedure, essentially as per the manufacturer’s protocol for the Qubit dsDNA HS assay kit. The quality and quantity of the isolated DNA was measured on a 0.8% agarose gel and Qubit dsDNA HS assay kit, respectively. The DNA fragmentation and library construction were done using a Nextera DNA Flex library preparation kit (Illumina) following manufacturers’ protocol. After library construction, dual index adapters were ligated at the blunt end of the DNA fragments. The quality and quantity of the fragment library were estimated and checked using the Qubit dsDNA HS assay kit and Agilent 2200 TapeStation, respectively. The good-quality library was normalized, pooled, and subsequently sequenced using 2 × 250-bp chemistry on a MiSeq platform (Illumina Inc., San Diego, CA, USA). The quality of the raw sequence was checked using FastQC (41). Adapter removal and trimming were done using Cutadapt (42).

Genome assembly and annotation were performed as previously reported (43). Assembly of trimmed good-quality reads was performed using the Unicycler assembler v.0.4.8 (44) in PATRIC (45). The quality of genome assembly was checked as per the QUAST (v.5.0.2) report (46). The assembled contig file was annotated using both the PATRIC and RAST servers (47). The annotation in PATRIC was performed using the default parameters, while that in RAST, the RASTtk pipeline (48), was used with few customizations. The quality of the annotated genome was assessed using the genome report file generated, based on which, further analyses were performed using PATRIC or RAST and other bioinformatic tools as per the requirements.

### Genome mining for CAZymes involved in polysaccharide degradation.

The CAZymes of *Paenibacillus* sp. LS1 were annotated using the dbCAN2 meta-server (49). An integrated approach using all the designated tools of the dbCAN2 meta-server, i.e., HMMER, Hotpep, Diamond, and CGC Finder was used for CAZyme prediction and annotation. Only those proteins or domains annotated by at least two tools were considered for further analysis (49).

CAZymes involved in the degradation of cellulose and xylan were identified with reference to the CAZy database (http://www.cazy.org/), and the protein sequences were retrieved from the annotated genome. The domain architecture of the CAZymes was predicted using Pfam (https://pfam.xfam.org/) and the NCBI Conserved Domain Database (https://www.ncbi.nlm.nih.gov/Structure/cdd/cdd.shtml). SignalP (v.5.0) (https://services.healthtech.dtu.dk/services/SignalP-5.0/) was used to predict the presence of signal peptide. The retrieved CAZymes were screened against the PDB database using blastp to understand the extent of their uniqueness compared to the already existing well-characterized counterparts. Furthermore, proteins involved in the transport and metabolism of these polysaccharides and their degradation products were also identified. This was implemented by performing a blastp search in PATRIC against the genome of *Paenibacillus* sp. LS1, using the amino acid sequence of a target protein as the query.

### Comparative genome analysis.

Genome-based relatedness between *Paenibacillus* sp. LS1 and its phylogenetic neighbors (7) was estimated by comparing the OrthoANIu and *in silico* DDH values. The ANI calculator of the EzBioCloud database (50) and Genome-to-Genome Distance Calculator (GGDC) of DSMZ (51) were used for calculating OrthoANIu and DDH values, respectively. The genomes of the phylogenetic neighbors used for estimating OrthoANIu and DDH values were restricted to the type strains only.

CAZymes involved in cellulose and xylan degradation were identified from the CAZy database, and CAZyme profiles were generated and compiled using the dbCAN2 meta-server for 238 genomes of distinct *Paenibacillus* species (including *Paenibacillus* sp. LS1). The *Paenibacillus* species were identified as per the List of Prokaryotic names with Standing in Nomenclature (LPSN) (52) and were selected based on genome availability in the NCBI genome database. A clustered heatmap of the predicted cellulose and xylan-active CAZyme profiles of 238 *Paenibacillus* species was constructed in R (v.4.1.2) using the package pheatmap (53). Log_10_-transformed values were plotted for easier visualization.

### RNA isolation, cDNA synthesis, and quantitative real-time PCR.

Total RNA was isolated from the harvested cells of *Paenibacillus* sp. LS1 grown up to the mid-log phase on different substrates (glucose, beechwood, and corncob xylan). Total RNA isolation was performed using the TRIzol method as per the manufacturer’s protocol (Sigma-Aldrich, USA). RNA integrity was analyzed using agarose gel electrophoresis and a NanoDrop 2000 UV-Vis spectrophotometer (Thermo Scientific, USA). Primers targeted for the selected genes involved in xylan degradation, xylooligosaccharides transport, and xylose metabolism were designed using the PrimerQuest tool of Integrated DNA Technologies (https://sg.idtdna.com/pages) and synthesized by Eurofins (Bengaluru, India) (Table S1). cDNA was synthesized from the total RNA using a PrimeScript 1st-strand cDNA synthesis kit (TaKaRa Bio, Inc., Japan). Quantitative real-time PCR was performed on a Mastercycler Realplex system (Eppendorf, Germany) in a final reaction volume of 10 μL containing 50 ng of cDNA, 0.4 μM primers, 1× SYBR TB green premix *Ex Taq* II (TliRNase H Plus), and 1× ROX reference dye I (6-carboxyX-rhodamine) (TaKaRa Bio, Inc.). The PCR conditions consisted of an initial denaturation at 95°C for 2 min, 40 cycles of amplification (95°C for 15 s, 54°C for 20 s, and 72°C for 30 s), and a final elongation stage at 72°C for 5 min. The gene *recA* (recombinase A) from *Paenibacillus* sp. LS1 was used as an internal control, and the relative fold change of RNA expression was estimated using the ΔΔ*C_T_* method (54).

### Substrate specificity of *Paenibacillus* sp. LS1 xylan-active secretomes.

Secretomes of *Paenibacillus* sp. LS1 grown in M9 medium supplemented with 0.5% beechwood and corncob xylan were collected to determine specificity toward different xylan substrates, while the secretome collected from glucose was considered a negative control. The bacteria were grown in the medium until the mid-log phase and were harvested by centrifugation at 5,000 rpm for 20 min at 4°C. Activity on beechwood and corncob xylans, wheat arabinoxylan, and 4-*O*-methyl-d-glucurono-d-xylan was determined using the DNS method as described in the section “Estimation of Extracellular Cellulase and Xylanase Activity.”. To determine activity on 4-nitrophenyl acetate, 100 μL of 2 mM 4-nitrophenyl acetate in 50 mM sodium phosphate, pH 7.0, was incubated with 100 μL of the secretome at 37°C for 10 min. The reaction was stopped by adding 200 μL of 1 M Na_2_CO_3_, and the absorbance was measured at 405 nm. The amount of 4-nitrophenol released was measured using the 4-nitrophenol standard curve. One unit of activity was defined as 1 μmol of 4-nitrophenol released per min.

### Data availability.

The genome of *Paenibacillus* sp. LS1 has been deposited at DDBJ/ENA/GenBank under accession no. JAPDOE000000000. The version described in this paper is version JAPDOE010000000.

## References

[1] Chundawat SP, Beckham GT, Himmel ME, Dale BE. 2011. Deconstruction of lignocellulosic biomass to fuels and chemicals. Annu Rev Chem Biomol Eng 2:121–145. doi:10.1146/annurev-chembioeng-061010-114205. 22432613

[2] Tõlgo M, Hegnar OA, Østby H, Várnai A, Vilaplana F, Eijsink VG, Olsson L. 2022. Comparison of six lytic polysaccharide monooxygenases from Thermothielavioides terrestris shows that functional variation underlies the multiplicity of LPMO genes in filamentous fungi. Appl Environ Microbiol 88:e00096-22. doi:10.1128/aem.00096-22. 35080911 PMC8939357

[3] Horn SJ, Vaaje-Kolstad G, Westereng B, Eijsink V. 2012. Novel enzymes for the degradation of cellulose. Biotechnol Biofuels 5:45–13. doi:10.1186/1754-6834-5-45. 22747961 PMC3492096

[4] Saha BC, Bothast RJ. 1999. Enzymology of xylan degradation. ACS Symp Ser 723:167–194. doi:10.1021/bk-1999-0723.ch011.

[5] Wu H, Ioannou E, Henrissat B, Montanier CY, Bozonnet S, O’Donohue MJ, Dumon C. 2021. Multimodularity of a GH10 xylanase found in the termite gut metagenome. Appl Environ Microbiol 87:e01714-20. doi:10.1128/AEM.01714-20. 33187992 PMC7848910

[6] Scheller HV, Ulvskov P. 2010. Hemicelluloses. Annu Rev Plant Biol 61:263–289. doi:10.1146/annurev-arplant-042809-112315. 20192742

[7] Mukherjee S, Behera PK, Madhuprakash J. 2020. Efficient conversion of crystalline chitin to N-acetylglucosamine and N,N′-diacetylchitobiose by the enzyme cocktail produced by Paenibacillus sp. LS1. Carbohydr Polym 250:116889. doi:10.1016/j.carbpol.2020.116889. 33049827

[8] Sanchez MM, Fritze D, Blanco A, Spröer C, Tindall BJ, Schumann P, Kroppenstedt RM, Diaz P, Pastor FJ. 2005. Paenibacillus barcinonensis sp. nov., a xylanase-producing bacterium isolated from a rice field in the Ebro River delta. Int J Syst Evol Microbiol 55:935–939. doi:10.1099/ijs.0.63383-0. 15774688

[9] Nelson DM, Glawe AJ, Labeda DP, Cann IK, Mackie RI. 2009. Paenibacillus tundrae sp. nov. and Paenibacillus xylanexedens sp. nov., psychrotolerant, xylan-degrading bacteria from Alaskan tundra. Int J Syst Evol Microbiol 59:1708–1714. doi:10.1099/ijs.0.004572-0. 19542122

[10] Ghio S, Sauka DH, Ferrari AE, Piccini FE, Ontañon OM, Campos E. 2019. Paenibacillus xylanivorans sp. nov., a xylan-degrading bacterium isolated from decaying forest soil. Int J Syst Evol Microbiol 69:3818–3823. doi:10.1099/ijsem.0.003686. 31483753

[11] Sainz-Polo MA, González B, Menéndez M, Pastor FJ, Sanz-Aparicio J. 2015. Exploring multimodularity in plant cell wall deconstruction: structural and functional analysis of Xyn10C containing the CBM22-1–CBM22-2 tandem. J Biol Chem 290:17116–17130. doi:10.1074/jbc.M115.659300. 26001782 PMC4498050

[12] Teeravivattanakit T, Baramee S, Phitsuwan P, Waeonukul R, Pason P, Tachaapaikoon C, Sakka K, Ratanakhanokchai K. 2016. Novel trifunctional xylanolytic enzyme Axy43A from Paenibacillus curdlanolyticus strain B-6 exhibiting endo-xylanase, β-D-xylosidase, and arabinoxylan arabinofuranohydrolase activities. Appl Environ Microbiol 82:6942–6951. doi:10.1128/AEM.02256-16. 27663030 PMC5103093

[13] Valenzuela SV, Lopez S, Biely P, Sanz-Aparicio J, Pastor FJ. 2016. The glycoside hydrolase family 8 reducing-end xylose-releasing exo-oligoxylanase Rex8A from Paenibacillus barcinonensis BP-23 is active on branched xylooligosaccharides. Appl Environ Microbiol 82:5116–5124. doi:10.1128/AEM.01329-16. 27316951 PMC4988184

[14] Jiménez-Ortega E, Valenzuela S, Ramírez-Escudero M, Pastor FJ, Sanz-Aparicio J. 2020. Structural analysis of the reducing-end xylose-releasing exo-oligoxylanase Rex8A from Paenibacillus barcinonensis BP-23 deciphers its molecular specificity. FEBS J 287:5362–5374. doi:10.1111/febs.15332. 32352213

[15] McLean JS, Lombardo M-J, Badger JH, Edlund A, Novotny M, Yee-Greenbaum J, Vyahhi N, Hall AP, Yang Y, Dupont CL, Ziegler MG, Chitsaz H, Allen AE, Yooseph S, Tesler G, Pevzner PA, Friedman RM, Nealson KH, Venter JC, Lasken RS. 2013. Candidate phylum TM6 genome recovered from a hospital sink biofilm provides genomic insights into this uncultivated phylum. Proc Natl Acad Sci U S A 110:E2390–E2399. doi:10.1073/pnas.1219809110. 23754396 PMC3696752

[16] St John FJ, Dietrich D, Crooks C, Pozharski E, González JM, Bales E, Smith K, Hurlbert JC. 2014. A novel member of glycoside hydrolase family 30 subfamily 8 with altered substrate specificity. Acta Crystallogr D Biol Crystallogr 70:2950–2958. doi:10.1107/S1399004714019531. 25372685 PMC4722856

[17] Nakamichi Y, Fouquet T, Ito S, Watanabe M, Matsushika A, Inoue H. 2019. Structural and functional characterization of a bifunctional GH30-7 xylanase B from the filamentous fungus Talaromyces cellulolyticus. J Biol Chem 294:4065–4078. doi:10.1074/jbc.RA118.007207. 30655295 PMC6422087

[18] Paës G, Skov LK, O’Donohue MJ, Rémond C, Kastrup JS, Gajhede M, Mirza O. 2008. The structure of the complex between a branched pentasaccharide and Thermobacillus xylanilyticus GH-51 arabinofuranosidase reveals xylan-binding determinants and induced fit. Biochemistry 47:7441–7451. doi:10.1021/bi800424e. 18563919

[19] Golan G, Shallom D, Teplitsky A, Zaide G, Shulami S, Baasov T, Stojanoff V, Thompson A, Shoham Y, Shoham G. 2004. Crystal structures of Geobacillus stearothermophilus α-glucuronidase complexed with its substrate and products: mechanistic implications. J Biol Chem 279:3014–3024. doi:10.1074/jbc.M310098200. 14573597

[20] Till M, Goldstone DC, Attwood GT, Moon CD, Kelly WJ, Arcus VL. 2013. Structure and function of an acetyl xylan esterase (Est2A) from the rumen bacterium Butyrivibrio proteoclasticus. Proteins 81:911–917. doi:10.1002/prot.24254. 23345031

[21] Schubot FD, Kataeva IA, Blum DL, Shah AK, Ljungdahl LG, Rose JP, Wang B-C. 2001. Structural basis for the substrate specificity of the feruloyl esterase domain of the cellulosomal xylanase Z from Clostridium thermocellum. Biochemistry 40:12524–12532. doi:10.1021/bi011391c. 11601976

[22] Gloster TM, Ibatullin FM, Macauley K, Eklöf JM, Roberts S, Turkenburg JP, Bjørnvad ME, Jørgensen PL, Danielsen S, Johansen KS, Borchert TV, Wilson KS, Brumer H, Davies GJ. 2007. Characterization and three-dimensional structures of two distinct bacterial xyloglucanases from families GH5 and GH12. J Biol Chem 282:19177–19189. doi:10.1074/jbc.M700224200. 17376777

[23] Murakami MT, Arni RK, Vieira DS, Degreve L, Ruller R, Ward RJ. 2005. Correlation of temperature induced conformation change with optimum catalytic activity in the recombinant G/11 xylanase A from Bacillus subtilis strain 168 (1A1). FEBS Lett 579:6505–6510. doi:10.1016/j.febslet.2005.10.039. 16289057

[24] Conway JM, Crosby JR, Hren AP, Southerland RT, Lee LL, Lunin VV, Alahuhta P, Himmel ME, Bomble YJ, Adams MWW, Kelly RM. 2018. Novel multidomain, multifunctional glycoside hydrolases from highly lignocellulolytic Caldicellulosiruptor species. AIChE J 64:4218–4228. doi:10.1002/aic.16354.

[25] Santos CR, Hoffmam ZB, de Matos Martins VP, Zanphorlin LM, de Paula Assis LH, Honorato RV, de Oliveira PSL, Ruller R, Murakami MT. 2014. Molecular mechanisms associated with xylan degradation by Xanthomonas plant pathogens. J Biol Chem 289:32186–32200. doi:10.1074/jbc.M114.605105. 25266726 PMC4231694

[26] Sainz-Polo MA, Valenzuela SV, González B, Pastor FJ, Sanz-Aparicio J. 2014. Structural analysis of glucuronoxylan-specific Xyn30D and its attached CBM35 domain gives insights into the role of modularity in specificity. J Biol Chem 289:31088–31101. doi:10.1074/jbc.M114.597732. 25202007 PMC4223313

[27] Czjzek M, David AB, Bravman T, Shoham G, Henrissat B, Shoham Y. 2005. Enzyme–substrate complex structures of a GH39 β-xylosidase from Geobacillus stearothermophilus. J Mol Biol 353:838–846. doi:10.1016/j.jmb.2005.09.003. 16212978

[28] Shulami S, Zaide G, Zolotnitsky G, Langut Y, Feld G, Sonenshein AL, Shoham Y. 2007. A two-component system regulates the expression of an ABC transporter for xylo-oligosaccharides in Geobacillus stearothermophilus. Appl Environ Microbiol 73:874–884. doi:10.1128/AEM.02367-06. 17142383 PMC1800775

[29] Li X, Chen Y, Nielsen J. 2019. Harnessing xylose pathways for biofuels production. Curr Opin Biotechnol 57:56–65. doi:10.1016/j.copbio.2019.01.006. 30785001

[30] Gu Y, Ding Y, Ren C, Sun Z, Rodionov DA, Zhang W, Yang S, Yang C, Jiang W. 2010. Reconstruction of xylose utilization pathway and regulons in Firmicutes. BMC Genomics 11:255–214. doi:10.1186/1471-2164-11-255. 20406496 PMC2873477

[31] Haddad Momeni M, Fredslund F, Bissaro B, Raji O, Vuong TV, Meier S, Nielsen TS, Lombard V, Guigliarelli B, Biaso F, Haon M, Grisel S, Henrissat B, Welner DH, Master ER, Berrin J-G, Abou Hachem M. 2021. Discovery of fungal oligosaccharide-oxidising flavo-enzymes with previously unknown substrates, redox-activity profiles and interplay with LPMOs. Nat Commun 12:2132. doi:10.1038/s41467-021-22372-0. 33837197 PMC8035211

[32] Ferrari AR, Rozeboom HJ, Dobruchowska JM, van Leeuwen SS, Vugts AS, Koetsier MJ, Visser J, Fraaije MW. 2016. Discovery of a xylooligosaccharide oxidase from Myceliophthora thermophila C1. J Biol Chem 291:23709–23718. doi:10.1074/jbc.M116.741173. 27629413 PMC5095424

[33] Grady EN, MacDonald J, Liu L, Richman A, Yuan Z-C. 2016. Current knowledge and perspectives of Paenibacillus: a review. Microb Cell Fact 15:203. doi:10.1186/s12934-016-0603-7. 27905924 PMC5134293

[34] López-Mondéjar R, Zühlke D, Větrovský T, Becher D, Riedel K, Baldrian P. 2016. Decoding the complete arsenal for cellulose and hemicellulose deconstruction in the highly efficient cellulose decomposer Paenibacillus O199. Biotechnol Biofuels 9:104. doi:10.1186/s13068-016-0518-x. 27186238 PMC4867992

[35] Ghio S, Insani EM, Piccinni FE, Talia PM, Grasso DH, Campos E. 2016. GH10 XynA is the main xylanase identified in the crude enzymatic extract of Paenibacillus sp. A59 when grown on xylan or lignocellulosic biomass. Microbiol Res 186–187:16–26. doi:10.1016/j.micres.2016.02.006. 27242139

[36] Vandhana TM, Reyre JL, Sushmaa D, Berrin JG, Bissaro B, Madhuprakash J. 2022. On the expansion of biological functions of lytic polysaccharide monooxygenases. New Phytol 233:2380–2396. doi:10.1111/nph.17921. 34918344

[37] Couturier M, Ladevèze S, Sulzenbacher G, Ciano L, Fanuel M, Moreau C, Villares A, Cathala B, Chaspoul F, Frandsen KE, Labourel A, Herpoël-Gimbert I, Grisel S, Haon M, Lenfant N, Rogniaux H, Ropartz D, Davies GJ, Rosso M-N, Walton PH, Henrissat B, Berrin J-G. 2018. Lytic xylan oxidases from wood-decay fungi unlock biomass degradation. Nat Chem Biol 14:306–310. doi:10.1038/nchembio.2558. 29377002

[38] Corrêa TLR, Júnior AT, Wolf LD, Buckeridge MS, Dos Santos LV, Murakami MT. 2019. An actinobacteria lytic polysaccharide monooxygenase acts on both cellulose and xylan to boost biomass saccharification. Biotechnol Biofuels 12:117. doi:10.1186/s13068-019-1449-0. 31168322 PMC6509861

[39] Sawhney N, Crooks C, St John F, Preston JF. 2015. Transcriptomic analysis of xylan utilization systems in Paenibacillus sp. strain JDR-2. Appl Environ Microbiol 81:1490–1501. doi:10.1128/AEM.03523-14. 25527555 PMC4309694

[40] Miller GL. 1959. Use of dinitrosalicylic acid reagent for determination of reducing sugar. Anal Chem 31:426–428. doi:10.1021/ac60147a030.

[41] Brown J, Pirrung M, McCue LA. 2017. FQC Dashboard: integrates FastQC results into a web-based, interactive, and extensible FASTQ quality control tool. Bioinformatics 33:3137–3139. doi:10.1093/bioinformatics/btx373. 28605449 PMC5870778

[42] Martin M. 2011. Cutadapt removes adapter sequences from high-throughput sequencing reads. EMBnet J 17:10–12. doi:10.14806/ej.17.1.200.

[43] Duhsaki L, Mukherjee S, Rani TS, Madhuprakash J. 2022. Genome analysis of Streptomyces sp. UH6 revealed the presence of potential chitinolytic machinery crucial for chitosan production. Environ Microbiol Rep 14:431–442. doi:10.1111/1758-2229.12986. 34192819

[44] Wick RR, Judd LM, Gorrie CL, Holt KE. 2017. Unicycler: resolving bacterial genome assemblies from short and long sequencing reads. PLoS Comput Biol 13:e1005595. doi:10.1371/journal.pcbi.1005595. 28594827 PMC5481147

[45] Wattam AR, Abraham D, Dalay O, Disz TL, Driscoll T, Gabbard JL, Gillespie JJ, Gough R, Hix D, Kenyon R, Machi D, Mao C, Nordberg EK, Olson R, Overbeek R, Pusch GD, Shukla M, Schulman J, Stevens RL, Sullivan DE, Vonstein V, Warren A, Will R, Wilson MJC, Yoo HS, Zhang C, Zhang Y, Sobral BW. 2014. PATRIC, the bacterial bioinformatics database and analysis resource. Nucleic Acids Res 42:D581–D591. doi:10.1093/nar/gkt1099. 24225323 PMC3965095

[46] Gurevich A, Saveliev V, Vyahhi N, Tesler G. 2013. QUAST: quality assessment tool for genome assemblies. Bioinformatics 29:1072–1075. doi:10.1093/bioinformatics/btt086. 23422339 PMC3624806

[47] Overbeek R, Olson R, Pusch GD, Olsen GJ, Davis JJ, Disz T, Edwards RA, Gerdes S, Parrello B, Shukla M, Vonstein V, Wattam AR, Xia F, Stevens R. 2014. The SEED and the Rapid Annotation of microbial genomes using Subsystems Technology (RAST). Nucleic Acids Res 42:D206–D214. doi:10.1093/nar/gkt1226. 24293654 PMC3965101

[48] Brettin T, Davis JJ, Disz T, Edwards RA, Gerdes S, Olsen GJ, Olson R, Overbeek R, Parrello B, Pusch GD, Shukla M, Thomason JA, Stevens R, Vonstein V, Wattam AR, Xia F. 2015. RASTtk: a modular and extensible implementation of the RAST algorithm for building custom annotation pipelines and annotating batches of genomes. Sci Rep 5:8365–8366. doi:10.1038/srep08365. 25666585 PMC4322359

[49] Zhang H, Yohe T, Huang L, Entwistle S, Wu P, Yang Z, Busk PK, Xu Y, Yin Y. 2018. dbCAN2: a meta server for automated carbohydrate-active enzyme annotation. Nucleic Acids Res 46:W95–W101. doi:10.1093/nar/gky418. 29771380 PMC6031026

[50] Yoon S-H, Ha S-M, Lim J, Kwon S, Chun J. 2017. A large-scale evaluation of algorithms to calculate average nucleotide identity. Antonie Van Leeuwenhoek 110:1281–1286. doi:10.1007/s10482-017-0844-4. 28204908

[51] Meier-Kolthoff JP, Auch AF, Klenk H-P, Göker M. 2013. Genome sequence-based species delimitation with confidence intervals and improved distance functions. BMC Bioinformatics 14:60–14. doi:10.1186/1471-2105-14-60. 23432962 PMC3665452

[52] Parte AC, Carbasse JS, Meier-Kolthoff JP, Reimer LC, Göker M. 2020. List of Prokaryotic names with Standing in Nomenclature (LPSN) moves to the DSMZ. Int J Syst Evol Microbiol 70:5607–5612. doi:10.1099/ijsem.0.004332. 32701423 PMC7723251

[53] Kolde R, Kolde MR. 2018. Package ‘pheatmap’. R Package 1. https://cran.r-project.org/web/packages/pheatmap/index.html.

[54] Livak KJ, Schmittgen TD. 2001. Analysis of relative gene expression data using real-time quantitative PCR and the 2–ΔΔCT method. Methods 25:402–408. doi:10.1006/meth.2001.1262. 11846609

