# Supplementary material for: Comprehensive Genome Analysis of Cellulose and Xylan-Active CAZymes from the Genus Paenibacillus: Special Emphasis on the Novel Xylanolytic Paenibacillus sp. LS1
Source: Microbiol Spectr. 2023 Apr 18;11(3):e05028-22. doi: 10.1128/spectrum.05028-22 (PMC10269863; doi:10.1128/spectrum.05028-22)
Supplement: Supplemental file 1 — Supplemental material. Download spectrum.05028-22-s0001.pdf, PDF file, 1.6 MB [file spectrum.05028-22-s0001.pdf]

Supplementary Information

Comprehensive genome analysis of cellulose and xylan-active CAZymes from the genus *Paenibacillus*: Special emphasis on the novel xylanolytic *Paenibacillus* sp. LS1

Saumashish Mukherjee[1], Tushar Dilipchand Lodha[2] and Jogi Madhuprakash*[1]

[1]Department of Plant Sciences, School of Life Sciences, University of Hyderabad, Gachibowli, Hyderabad, India.

[2]National Centre for Microbial Resource, National Centre for Cell Science, Pune, India.

***Author for correspondence:**
Dr. Jogi Madhuprakash
Department of Plant Sciences
School of Life Sciences
University of Hyderabad
Prof. CR Rao Road, Gachibowli,
Hyderabad-500046, India
Tel: +91-40-23134566
Fax: +91-40-23010120
E-mail: jmpsl@uohyd.ac.in

Supplementary Information

Table S1: List of primers used for the qRT-PCR analysis of selected xylan-active genes from *Paenibacillus* sp. LS1.

S.No.	Name of the gene	Template used for qRT-PCR	Primer combination used	Primer sequence (5'- 3')
1.	peg.181	cDNA	peg181-Fp	GGTTCCGCAAGGATCAAT
			peg181-Rp	GATGCAGTGAAGGTTCGT
2.	peg.315	cDNA	peg315-Fp	ATGTTCAGGATGCGAAGG
			peg315-Rp	GTCGGTCATCTGCTTCATAG
3.	peg.184	cDNA	peg184-Fp	TAGACAGTCCAAGAGACC
			peg184-Rp	GACCAACTACTTCCAAGG
4.	peg.1758	cDNA	peg1758-Fp	CGCACCAGAAGAATACAG
			peg1758-Rp	CGATTACTCCCACCATAC
5.	peg.306	cDNA	peg306-Fp	GACAGAAGACCTGGATTC
			peg306-Rp	GGGTATGGATGAGTTGAG
6.	peg.1759	cDNA	peg.1759-Fp	GGTTCATGGCGAATCTAC
			peg1759-Rp	GGTAAGACCTGCAATCTC
7.	peg.5152	cDNA	peg5152-Fp	CGCTCAGTACAGACTTTG
			peg5152-Rp	CCAGTTCTCATTGCCTAC
8.	peg.549	cDNA	peg549-Fp	CAGGAGAGAATGGGTTTG
			peg549-Rp	GTCAGAGCTGTCGTAATG
9.	peg.1599	cDNA	peg1599-Fp	GAGCAAGACCTACTCAAG
			peg.1599-Rp	CACCTCCCATAGACAATC
10.	peg.3462	cDNA	peg3462-Fp	GAGCGTACAAGCGAATAC
			peg3462-Rp	GCGATAACTCTCCAGTTG
11.	peg.3263	cDNA	peg3263-Fp	GGGCGTATATGGTTGTTC
			peg3263-Rp	GAGTGTCTGTAGTCCGAT
12.	peg.3234	cDNA	peg3234-Fp	CCTTTGTCAGTCATCCTG
			peg3234-Rp	GAAGCGCGTAGTAGAATC
13.	peg.3235	cDNA	peg3235-Fp	GAGCCTCATCGGTTATCT
			peg3235-Rp	CAACAGCATCGGGATTAG
14.	peg.3236	cDNA	peg3236-Fp	GGGTTGTCTAACGATGAG
			peg3236-Rp	GCGGAATATCAGGATTGG
15.	peg.4642	cDNA	peg4642-Fp	GTCTCGTGGAATCGTTTG
			peg4642-Rp	CCGCCTGCATATACTTTC
16.	peg.4644	cDNA	peg4644-Fp	CTTCCCTTGCTGAGTATG
			peg4644-Rp	CAGGATGTACTGGTTGAG

Supplementary Information

Table S2: Conserved single copy gene list (107) and their presence within the genome of *Paenibacillus* sp. LS1.

S.no	Conserved genes	Homologue ID (PATRIC)	Contig	Strand	Start	End
1	cgtA	peg.5413	contig_10	(+)	217771	219081
2	coaE	peg.2656	contig_2	(-)	756849	757445
3	fmt	peg.1257	contig_1	(-)	1409599	1410552
4	ligA	peg.5869	contig_14	(-)	2232	4253
5	pgk	peg.5830	contig_13	(-)	95397	96578
6	pheT	peg.2894	contig_2	(-)	1025635	1028088
7	proS	peg.3724	contig_4	(+)	142238	144022
		peg.2324	contig_2	(-)	395242	396696
8	rnc	peg.2403	contig_2	(-)	471301	472005
9	rpoC	peg.6385	contig_20	(+)	28567	32181
10	secE	peg.6377	contig_20	(+)	20596	20787
11	dnaK	peg.1494	contig_1	(-)	1653782	1655626
12	ffh	peg.2398	contig_2	(-)	463388	464767
13	glyS	peg.1454	contig_1	(-)	1610480	1612555
14	gmk	peg.1262	contig_1	(-)	1415586	1416158
15	gyrB	peg.3596	contig_3	(+)	423548	425458
16	ksgA	peg.6257	contig_18	(+)	18160	19062
17	uvrB	peg.5884	contig_14	(-)	26880	28871
18	prfA	peg.4549	contig_6	(+)	260569	261636
19	alaS	peg.1941	contig_1	(-)	2148996	2151617
		peg.6086	contig_16	(-)	3896	5074
20	argS	peg.2265	contig_2	(-)	334966	336696
		peg.6508	contig_22	(+)	32115	33794
21	aspS	peg.6461	contig_21	(+)	28369	30147
22	cysS	peg.6372	contig_20	(+)	16114	17514
23	dnaA	peg.3591	contig_3	(+)	419030	420376
24	dnaG	peg.1452	contig_1	(-)	1607915	1609735
25	dnaN	peg.3592	contig_3	(+)	420597	421739
26	dnaX	peg.6522	contig_22	(+)	45517	47262
27	engA	peg.1006	contig_1	(-)	1134416	1135738
28	era	peg.1459	contig_1	(-)	1614877	1615776
29	frr	peg.2329	contig_2	(-)	401026	401580
30	ftsY	peg.2401	contig_2	(-)	466238	467251
31	grpE	peg.1495	contig_1	(-)	1655788	1656351
32	gyrA	peg.3598	contig_3	(+)	426921	429491
33	hisS	peg.6460	contig_21	(+)	26912	28165
34	ileS	peg.1389	contig_1	(-)	1539052	1542150
35	infB	peg.2318	contig_2	(-)	385599	388184
36	infC	peg.2920	contig_2	(-)	1057883	1058413
		peg.2080	contig_2	(-)	161379	161870
37	lepA	peg.1499	contig_1	(-)	1659704	1661518

38	leuS	peg.1518	contig_1	(-)	1678655	1681099
39	mnmA	peg.1948	contig_1	(+)	2156327	2157451
40	mraW	peg.1414	contig_1	(-)	1568884	1569834
41	nusA	peg.2321	contig_2	(-)	388830	389927
42	nusG	peg.6378	contig_20	(+)	20809	21342
43	pheS	peg.2895	contig_2	(-)	1028178	1029212
44	rpsT	peg.1503	contig_1	(+)	1664693	1664965
45	secA	peg.5915	contig_14	(-)	63058	65562
46	secG	peg.5826	contig_13	(-)	91093	91326
47	secY	peg.6412	contig_20	(+)	47066	48364
48	serS	peg.6477	contig_22	(+)	4732	6015
		peg.4945	contig_8	(+)	118309	119607
49	smpB	peg.5823	contig_13	(-)	87006	87488
50	thrS	peg.3208	contig_2	(+)	1304937	1306949
		peg.2801	contig_2	(-)	922474	924411
51	tig	peg.5377	contig_10	(+)	177980	179320
52	tilS	peg.6285	contig_18	(+)	44732	46171
53	tsf	peg.2331	contig_2	(-)	402540	403190
54	tyrS	peg.776	contig_1	(-)	899154	900416
55	valS	peg.5394	contig_10	(+)	199488	202154
56	ybeY	peg.1462	contig_1	(-)	1616911	1617408
57	ychF	peg.4547	contig_6	(+)	257956	259056
58	pyrG	peg.6513	contig_22	(+)	36723	38327
59	recA	peg.2288	contig_2	(-)	354710	355774
60	rfbA	peg.5277	contig_10	(+)	65761	66492
61	rplA	peg.6380	contig_20	(+)	21931	22623
62	rplB	peg.6395	contig_20	(+)	39569	40399
63	rplC	peg.6392	contig_20	(+)	37976	38599
64	rplD	peg.6393	contig_20	(+)	38625	39248
65	rplE	peg.6404	contig_20	(+)	43567	44109
66	rplF	peg.6407	contig_20	(+)	44830	45372
67	rplI	peg.3549	contig_3	(-)	378867	379310
68	rplJ	peg.6381	contig_20	(+)	22862	23365
69	rplK	peg.6379	contig_20	(+)	21411	21836
70	rplL	peg.6382	contig_20	(+)	23438	23797
71	rplM	peg.6423	contig_20	(+)	54085	54522
72	rplN	peg.6402	contig_20	(+)	42773	43141
73	rplO	peg.6411	contig_20	(+)	46626	47066
74	rplP	peg.6399	contig_20	(+)	41801	42235
75	rplQ	peg.6421	contig_20	(+)	52579	52944
76	rplR	peg.6408	contig_20	(+)	45490	45858
77	rplS	peg.2389	contig_2	(-)	457565	457906
78	rplT	peg.2918	contig_2	(-)	1057207	1057566
79	rplU	peg.5409	contig_10	(+)	215875	216186
80	rplV	peg.6397	contig_20	(+)	40785	41117
81	rplW	peg.6394	contig_20	(+)	39248	39538

82	rplX	peg.6403	contig_20	(+)	43181	43534
83	rpmA	peg.5411	contig_10	(+)	216554	216865
84	rpmB	peg.1248	contig_1	(+)	1401298	1401486
85	rpmC	peg.6400	contig_20	(+)	42225	42422
86	rpmF	peg.2411	contig_2	(-)	478702	478875
87	rpmH	peg.3585	contig_3	(-)	413587	413721
88	rpmI	peg.2919	contig_2	(-)	1057655	1057858
89	rpoA	peg.6420	contig_20	(+)	51600	52544
90	rpoB	peg.6384	contig_20	(+)	24908	28453
91	rpsB	peg.2332	contig_2	(-)	403306	404004
92	rpsC	peg.6398	contig_20	(+)	41133	41798
93	rpsD	peg.1287	contig_1	(+)	1444409	1445008
94	rpsE	peg.6409	contig_20	(+)	45886	46383
95	rpsF	peg.3567	contig_3	(-)	398279	398572
96	rpsG	peg.6388	contig_20	(+)	33300	33770
97	rpsH	peg.6406	contig_20	(+)	44401	44799
98	rpsI	peg.6424	contig_20	(+)	54542	54934
99	rpsJ	peg.6391	contig_20	(+)	37621	37929
100	rpsK	peg.6419	contig_20	(+)	51068	51463
101	rpsL	peg.6387	contig_20	(+)	32790	33212
102	rpsM	peg.6418	contig_20	(+)	50680	51048
103	rpsO	peg.2313	contig_2	(-)	381863	382132
104	rpsP	peg.2397	contig_2	(-)	463063	463335
105	rpsQ	peg.6401	contig_20	(+)	42471	42737
106	rpsR	peg.3565	contig_3	(-)	397414	397686
107	rpsS	peg.6396	contig_20	(+)	40471	40749

Supplementary Information

Table S3: Represents the presence or absence of signal peptide and sub-cellular localization of the xylan degrading/modifying enzymes of *Paenibacillus* sp. LS1. The Sequence ID is as per genome annotation in PATRIC. The CAZy family of the respective enzymes have been determined by dbCAN2 meta server. Signal peptide prediction was done using SignalP 5.0. The prediction Sec/SPI stands for "standard" secretory signal peptides transported by the Sec translocon and cleaved by Signal Peptidase I (Lep).

Sequence ID (PATRIC)	Family	Annotation	Signal peptide	SignalP prediction
peg.2174	GH5	Endo-1,4-β-xylanase	Yes	Sec/SPI
peg.181	GH8	Oligosaccharide reducing-end xylanase	No	None
peg.315	GH10	Endo-1,4-β-xylanase	Yes	Sec/SPI
peg.1826	GH10	Endo-1,4-β-xylanase	No	None
peg.2768	GH10	Endo-1,4-β-xylanase	Yes	Sec/SPI
peg.3441	GH10	Xylanase	Yes	Sec/SPI
peg.184	GH11	Endo-1,4-β-xylanase	Yes	Sec/SPI
peg.1758	GH30	Hypothetical protein (α-L-arabinofuranosidase B)	Yes	Sec/SPI
peg.1761	GH30	α-L-arabinofuranosidase B	No	None
peg.2541	GH30	Glucuronoarabinoxylan endo-1,4-β-xylanase	Yes	Sec/SPI
peg.306	GH39	Xylan 1,4-β-xylosidase	No	None
peg.159	GH43	Arabinoxylan arabinofuranohydrolase	No	None
peg.507	GH43	α-L-arabinofuranosidase II	No	None
peg.1759	GH43	Hypothetical protein (Putative arabinofuranosidase)	Yes	Sec/SPI
peg.3708	GH43	α-L-arabinofuranosidase II	No	None
peg.161	GH43	Possible β-xylosidase	No	None
peg.308	GH43	Xylan 1,4-β-xylosidase	No	None
peg.1836	GH43	Xylan 1,4-β-xylosidase	No	None

peg.3696	GH43	Xylan 1,4-β-xylosidase	No	None
peg.5350	GH43	Xylan 1,4-β-xylosidase	No	None
peg.435	GH43	Hypothetical protein	No	None
peg.253	GH51	α-L-arabinofuranosidase	No	None
peg.3680	GH51	α-L-arabinofuranosidase	No	None
peg.5152	GH51	α-L-arabinofuranosidase	No	None
peg.6505	GH51	α-L-arabinofuranosidase	No	None
peg.549	GH67	Xylan α-1,2-glucuronosidase	No	None
peg.183	CE1	Endo-1,4-β-xylanase (Feruloyl Esterase)	No	None
peg.1599	CE1	Endo-1,4-β-xylanase (Feruloyl Esterase)	Yes	Sec/SPI
peg.3462	CE2	Esterase	No	None
peg.3263	CE7	Acetyl xylan esterase	No	None

Supplementary Information

Table S4: Represents the presence or absence of signal peptide and sub-cellular localization of the cellulose degrading enzymes of *Paenibacillus* sp. LS1. The Sequence ID is as per genome annotation in PATRIC. The CAZy family of the respective enzymes have been determined by dbCAN2 meta server. Signal peptide prediction was done using SignalP 5.0. The prediction Sec/SPI stands for "standard" secretory signal peptides transported by the Sec translocon and cleaved by Signal Peptidase I (Lep).

Sequence ID (PATRIC)	Family	Annotation	Signal peptide	SignalP prediction
peg.4664	GH1	β-glucosidase	No	None
peg.1242	GH3	β-glucosidase	Yes	Sec/SPI
peg.2464	GH3	β-glucosidase	No	None
peg.2780	GH3	β-glucosidase	No	None
peg.4423	GH3	β-glucosidase	No	None
peg.5236	GH3	β-glucosidase	No	None
peg.5446	GH3	β-glucosidase	No	None
peg.5760	GH3	β-glucosidase	No	None
peg.6114	GH3	β-glucosidase	Yes	Sec/SPI
peg.6312	GH3	β-glucosidase	No	None
peg.5091	GH5	Endo-1,4-β-glucanase (Cellulase B)	Yes	Sec/SPI
peg.5652	GH6	Cellulase	Yes	Sec/SPI
peg.3676	GH9	β-1,4-glucanase (cellulase)	Yes	Sec/SPI
peg.3677	GH48	Cellulose 1,4-β-cellobiosidase	Yes	Sec/SPI

Supplementary Information

Table S5: *in silico* OrthoANIu and DDH values for *Paenibacillus* sp. LS1 compared against its phylogenetic neighbours

**Phylogenetic neighbours**	**OrthoANIu**	**DDH**
Paenibacillus amylolyticus NBRC 15957	92.17%	46.90%
Paenibacillus xylanexedens DSM 21292	92.28%	47.10%
*Paenibacillus polysaccharolyticus*BL9	78.53%	22.50%
Paenibacillus pabuli NBRC 13638	81.87%	25.20%
Paenibacillus xylanilyticus LMG 21957	80.60%	23.90%
Paenibacillus taichungensis DSM 19942	82.07%	25.50%

Supplementary Information

Table S6: List of *Paenibacillus* genomes used for the comparative genome analysis

S.no.	Species	Accession	Assembly Level
1	*Paenibacillus agaridevorans* HCL2020	NZ_CP075889.1	Complete
2	*Paenibacillus albicereus* UniB2	NZ_CP051428.1	Complete
3	*Paenibacillus albus* 18JY67-1	NZ_CP034437.1	Complete
4	*Paenibacillus algicola* HB172198	NZ_CP040396.1	Complete
5	*Paenibacillus azotofixans* ATCC 35681	NZ_CP011114.1	Complete
6	*Paenibacillus baekrokdamisoli* KCTC 33723	NZ_AP019308.1	Complete
7	*Paenibacillus beijingensis* DSM 24997	NZ_CP011058.1	Complete
8	*Paenibacillus borealis* DSM 13188	NZ_CP009285.1	Complete
9	*Paenibacillus bovis* BD3526	NZ_CP013023.1	Complete
10	*Paenibacillus brasilensis* KACC 13842	NZ_CP045298.1	Complete
11	*Paenibacillus cellulositrophicus* KACC 16577	NZ_CP045295.1	Complete
12	*Paenibacillus crassostreae* LPB0068	NZ_CP017770.1	Complete
13	*Paenibacillus donghaensis* KCTC 13049	NZ_CP021780.1	Complete
14	*Paenibacillus durus* DSM 1735	NZ_CP009288.1	Complete
15	*Paenibacillus graminis* DSM 15220	NZ_CP009287.1	Complete
16	*Paenibacillus guangzhouensis* KCTC 33171	NZ_CP045293.1	Complete
17	*Paenibacillus ihbetae* IHBB 9852	CP016809.1	Complete
18	*Paenibacillus kribbensis* AM49	NZ_CP020028.1	Complete
19	*Paenibacillus larvae* subsp. *larvae* strain ATCC 9545	NZ_CP019687.1	Complete
20	*Paenibacillus lentus* DSM 25539	NZ_CP034248.1	Complete
21	*Paenibacillus lutimineralis* MBLB1234	NZ_CP034346.1	Complete
22	*Paenibacillus lycopersici* 12200R-189	NZ_CP048209.1	Complete
23	*Paenibacillus mucilaginosus* K02	NC_017672.3	Complete
24	*Paenibacillus odorifer* DSM 15391	NZ_CP009428.1	Complete
25	*Paenibacillus peoriae* ZF390	NZ_CP061172.1	Complete
26	*Paenibacillus physcomitrellae* XB	NZ_CP022584.1	Complete
27	*Paenibacillus polymyxa* DSM 36	NZ_CP049783.1	Complete
28	*Paenibacillus protaetiae* FW100M-2	NZ_CP035492.1	Complete
29	*Paenibacillus psychroresistens* ML311-T8	NZ_CP034235.1	Complete
30	*Paenibacillus rhizovicinus* 14171R-81	NZ_CP048286.1	Complete

31	*Paenibacillus riograndensis* SBR5(T)	NZ_LN831776.1	Complete
32	*Paenibacillus sabinae* T27	NZ_CP004078.1	Complete
33	*Paenibacillus sonchi* LMG 24727	NZ_CP068595.1	Complete
34	*Paenibacillus sophorae* DSM 23020	NZ_CP076607.1	Complete
35	*Paenibacillus stellifer* DSM 14472	NZ_CP009286.1	Complete
36	*Paenibacillus swuensis* DY6	NZ_CP011388.1	Complete
37	*Paenibacillus terrae* HPL-003	NC_016641.1	Complete
38	*Paenibacillus thiaminolyticus* NRRL B-4156	NZ_CP041405.1	Complete
39	*Paenibacillus tianjinensis* TB2019	NZ_CP070969.1	Complete
40	*Paenibacillus uliginis* N3/975	NZ_LT840184.1	Complete
41	*Paenibacillus yonginensis* DCY84	NZ_CP014167.1	Complete
42	*Paenibacillus aceti* CGMCC 1.15420	BMIW00000000.1	Scaffold
43	*Paenibacillus alba* LMG 31323	NZ_JABMKZ000000000.1	Scaffold
44	*Paenibacillus alginolyticus* DSM 5050	AUGY00000000.1	Scaffold
45	*Paenibacillus algorifonticola* XJ259	LAQO00000000.1	Scaffold
46	*Paenibacillus allorhizosphaerae* CIP 111802	NZ_CAJVCE000000000.1	Scaffold
47	*Paenibacillus anaericanus* DSM 15890	NZ_RZNY00000000.1	Scaffold
48	*Paenibacillus anseongense* MAH-34	NZ_WSEM00000000.1	Scaffold
49	*Paenibacillus antibioticophila* GD11	CBLK000000000.1	Scaffold
50	*Paenibacillus arenosi* CAU 1523	NZ_JACYTN000000000.1	Scaffold
51	*Paenibacillus assamensis* DSM 18201	NZ_AULU00000000.1	Scaffold
52	*Paenibacillus azotifigens* LMG 29963	NZ_VWOY00000000.1	Scaffold
53	*Paenibacillus barcinonensis* CECT 7022	QJSW00000000.1	Scaffold
54	*Paenibacillus bouchesdurhonensis* Marseille-P3071	NZ_FTLT00000000.1	Scaffold
55	*Paenibacillus brevis* MSJ-6	NZ_JAHLQJ000000000.1	Scaffold
56	*Paenibacillus castaneae* DSM 19417	NJAR00000000.1	Scaffold
57	*Paenibacillus catalpae* CGMCC 1.10784	FOMT00000000.1	Scaffold
58	*Paenibacillus cellulosilyticus* CECT 5696	QGTQ00000000.1	Scaffold
59	*Paenibacillus contaminans* CKOBP-6	NZ_QMFB00000000.1	Scaffold
60	*Paenibacillus curdlanolyticus* YK9	AEDD00000000.1	Scaffold
61	*Paenibacillus daejeonensis* DSM 15491	NZ_ARKE00000000.1	Scaffold
62	*Paenibacillus darwinianus* Br	JFHT00000000.1	Scaffold

63	*Paenibacillus dauci* H9	NZ_LAQQ00000000.1	Scaffold
64	*Paenibacillus dokdonensis* YH-JAE5	NZ_STGQ00000000.1	Scaffold
65	*Paenibacillus endophyticus* CECT 8234	NZ_JACHXW000000000.1	Scaffold
66	*Paenibacillus etheri* SH7	NZ_LCZJ00000000.2	Scaffold
67	*Paenibacillus faecalis* Marseille-P3787	NZ_OKQQ00000000.1	Scaffold
68	*Paenibacillus faecis* DSM 23593	VSDO00000000.1	Scaffold
69	*Paenibacillus farraposensis* UY79	NZ_JAFFQR000000000.1	Scaffold
70	*Paenibacillus ferrarius* CY1	NZ_MBTG00000000.1	Scaffold
71	*Paenibacillus fonticola* DSM 21315	NZ_ARMT00000000.1	Scaffold
72	*Paenibacillus frigoriresistens* LMG 31322	NZ_JABRUY000000000.1	Scaffold
73	*Paenibacillus ginsengarvi* KCTC 13059	NZ_RBAH00000000.1	Scaffold
74	*Paenibacillus ginsengihumi* DSM 21568	NZ_ARKW00000000.1	Scaffold
75	*Paenibacillus harenae* DSM 16969	NZ_AULV00000000.1	Scaffold
76	*Paenibacillus hemerocallicola* KCTC 33185	NZ_VDCQ00000000.1	Scaffold
77	*Paenibacillus herberti* R33	NZ_NMUQ00000000.1	Scaffold
78	*Paenibacillus hunanensis* CGMCC 1.8907	NZ_BMMB00000000.1	Scaffold
79	*Paenibacillus ihumii* AT5	CYXK00000000.1	Scaffold
80	*Paenibacillus ihuae* GD6	CTED00000000.1	Scaffold
81	*Paenibacillus jilunlii* CGMCC 1.10239	FNGM00000000.1	Scaffold
82	*Paenibacillus lupini* CECT 8235	NZ_JAASRW000000000.1	Scaffold
83	*Paenibacillus luteus* R-3	NZ_SKCD00000000.1	Scaffold
84	*Paenibacillus lutrae* N10	RHLK00000000.1	Scaffold
85	*Paenibacillus macquariensis* ATCC 23464	FTNK00000000.1	Scaffold
86	*Paenibacillus marchantiophytorum* CGMCC 1.15043	NZ_BMHE00000000.1	Scaffold
87	*Paenibacillus massiliensis* DSM 16942	ARIL00000000.1	Scaffold
88	*Paenibacillus mesophilus* SYSU K30004	NZ_VCRA00000000.1	Scaffold
89	*Paenibacillus methanolicus* BL24	NZ_VNHS00000000.1	Scaffold
90	*Paenibacillus montaniterrae* J40TS1	NZ_BOSE00000000.1	Scaffold
91	*Paenibacillus oralis* KCOM 3021	NZ_RRCN00000000.1	Scaffold
92	*Paenibacillus oryzae* 1DrF-4	NZ_LYPA00000000.1	Scaffold
93	*Paenibacillus oryzisoli* 1ZS3-15	NZ_LYPB00000000.1	Scaffold
94	*Paenibacillus panacisoli* DSM 21345	NZ_AUFO00000000.1	Scaffold
95	*Paenibacillus paridis* py1325	NZ_VCIX00000000.1	Scaffold

96	*Paenibacillus pasadenensis* DSM 19293	AULW00000000.1	Scaffold
97	*Paenibacillus pectinilyticus* KCTC13222	NZ_LYPC00000000.1	Scaffold
98	*Paenibacillus phyllosphaerae* CECT 5862	NZ_JACHXK000000000.1	Scaffold
99	*Paenibacillus pinihumi* DSM 23905	AULX00000000.1	Scaffold
100	*Paenibacillus piscarius* P121	NZ_JAIEUI000000000.1	Scaffold
101	*Paenibacillus polysaccharolyticus* BL9	NZ_FMVM00000000.1	Scaffold
102	*Paenibacillus prosopidis* CECT 7506	NZ_QPJD00000000.1	Scaffold
103	*Paenibacillus puerhi* SJY2	NZ_WUWM00000000.1	Scaffold
104	*Paenibacillus qinlingensis* LMG 31363	NZ_JABMKY000000000.1	Scaffold
105	*Paenibacillus radicis* CGMCC 1.15286	NZ_BMHY00000000.1	Scaffold
106	*Paenibacillus rhizosphaerae* CECT 5831	JACHXJ000000000.1	Scaffold
107	*Paenibacillus roseus* MAHUQ-46	NZ_JAELUP000000000.1	Scaffold
108	*Paenibacillus rubinfantis* MT18	FAUQ00000000.1	Scaffold
109	*Paenibacillus sanguinis* DSM 16941	NZ_ARGO00000000.1	Scaffold
110	*Paenibacillus segetis* CGMCC 1.12769	NZ_BMFT00000000.1	Scaffold
111	*Paenibacillus senegalensis* JC66	CAES00000000.1	Scaffold
112	*Paenibacillus senegalimassiliensis* SIT18	FAUP00000000.1	Scaffold
113	*Paenibacillus silvae* CGMCC 1.12770	BMFU00000000.1	Scaffold
114	*Paenibacillus silvestris* 5-J6	NZ_WTUZ00000000.1	Scaffold
115	*Paenibacillus sinensis* HN-1	NZ_JAHCMB000000000.1	Scaffold
116	*Paenibacillus sinopodophylli* CCTCC AB 2016047	NZ_VDBO00000000.1	Scaffold
117	*Paenibacillus solani* FJAT-22460	LIUT00000000.1	Scaffold
118	*Paenibacillus taihuensis* CGMCC 1.10966	NZ_QTTN00000000.1	Scaffold
119	*Paenibacillus taiwanensis* DSM 18679	NZ_AULE00000000.1	Scaffold
120	*Paenibacillus tengchongensis* SYSU G01003	WACP00000000.1	Scaffold
121	*Paenibacillus terrigena* DSM 21567	ARGP00000000.1	Scaffold
122	*Paenibacillus thermoaerophilus* DSM 26310	VCQZ00000000.1	Scaffold
123	*Paenibacillus thermophilus* DSM 24746	BGML00000000.1	Scaffold
124	*Paenibacillus tianmuensis* CGMCC 1.8946	FMTT00000000.1	Scaffold

125	*Paenibacillus tritici* LMG 29502	JABMKX000000000.1	Scaffold
126	*Paenibacillus tuaregi* Marseille-P2472	FLKE00000000.1	Scaffold
127	*Paenibacillus typhae* CGMCC 1.11012	NZ_FNDX00000000.1	Scaffold
128	*Paenibacillus vini* J42TS3	BOSL00000000.1	Scaffold
129	*Paenibacillus woosongensis* J15TS10	BOSM00000000.1	Scaffold
130	*Paenibacillus wulumuqiensis* Y24	LAQP00000000.1	Scaffold
131	*Paenibacillus xylanivorans* A59	LITU00000000.1	Scaffold
132	*Paenibacillus abyssi* CGMCC 1.12987	BMGR00000000.1	Contig
133	*Paenibacillus aceris* DSM 24950	JAGGKV000000000.1	Contig
134	*Paenibacillus agri* JW14	NZ_JABWCS000000000.1	Contig
135	*Paenibacillus albidus* CGMCC 1.16134	BMKR00000000.1	Contig
136	*Paenibacillus albiflavus* 18JY21-1	NZ_SKFG00000000.1	Contig
137	*Paenibacillus albilobatus* J2TS6	NZ_BORQ00000000.1	Contig
138	*Paenibacillus alvei* DSM 29	NZ_AMBZ00000000.1	Contig
139	*Paenibacillus amylolyticus* NBRC 15957	BIMJ00000000.1	Contig
140	*Paenibacillus antarcticus* CECT 5836	LVJI00000000.1	Contig
141	*Paenibacillus antri* SYSU K30003	NZ_VCIW00000000.1	Contig
142	*Paenibacillus apiarius* MW-14	JAFFHZ000000000.1	Contig
143	*Paenibacillus apii* 7124	JAAKGU000000000.1	Contig
144	*Paenibacillus apis* J41TS4	BORS00000000.1	Contig
145	*Paenibacillus aquistagni* 11	FXAZ00000000.1	Contig
146	*Paenibacillus artemisiicola* MWE-103	NZ_JAGGDJ000000000.1	Contig
147	*Paenibacillus athensensis* MEC069	NZ_MYFO00000000.2	Contig
148	*Paenibacillus azoreducens* J34TS1	NZ_BORT00000000.1	Contig
149	*Paenibacillus barengoltzii* NBRC 101215	BILV00000000.1	Contig
150	*Paenibacillus camerounensis* G4	NZ_CCDG000000000.1	Contig
151	*Paenibacillus campinasensis* 7537-G1	NPBY00000000.1	Contig
152	*Paenibacillus caui* 81-11	NZ_JAIOGQ000000000.1	Contig
153	*Paenibacillus chibensis* NBRC 15958	NZ_BIMK00000000.1	Contig
154	*Paenibacillus chitinolyticus* NBRC 15660	BBJT00000000.1	Contig
155	*Paenibacillus chondroitinus* NBRC 15376	BILW00000000.1	Contig
156	*Paenibacillus cineris* J43TS9	BORV00000000.1	Contig
157	*Paenibacillus cisolokensis* LC2-13A	NZ_BOVJ00000000.1	Contig

158	*Paenibacillus cookii* J21TS3	NZ_BORW00000000.1	Contig
159	*Paenibacillus cucumis* S/N-304-OC-R4	NZ_JACLIC000000000.1	Contig
160	*Paenibacillus cymbidii* R196	NZ_SHLX00000000.1	Contig
161	*Paenibacillus dakarensis* FF9	NZ_CDSE00000000.1	Contig
162	*Paenibacillus dendritiformis* C454	AHKH00000000.1	Contig
163	*Paenibacillus ehimensis* NBRC 15659	BILX00000000.1	Contig
164	*Paenibacillus elgii* NBRC 100335	NZ_BIMB00000000.1	Contig
165	*Paenibacillus eucommiae* DSM 26048	NZ_JAGGLB000000000.1	Contig
166	*Paenibacillus favisporus* Y7	NZ_WIBG00000000.1	Contig
167	*Paenibacillus flagellatus* DXL2	NZ_QJVJ00000000.1	Contig
168	*Paenibacillus foliorum* LMG 31456	NZ_WHOD00000000.1	Contig
169	*Paenibacillus forsythiae* T98	NZ_ASSC00000000.1	Contig
170	*Paenibacillus germinis* LMG 31460	NZ_WHOC00000000.1	Contig
171	*Paenibacillus glacialis* DSM 22343	NZ_LVJH00000000.1	Contig
172	*Paenibacillus glucanolyticus* NBRC 15330	BIMC00000000.1	Contig
173	*Paenibacillus glycanilyticus* NBRC 16618	BILY00000000.1	Contig
174	*Paenibacillus glycinis* T1	NZ_JAAAMV000000000.1	Contig
175	*Paenibacillus gorillae* G1	NZ_CBVJ000000000.1	Contig
176	*Paenibacillus helianthi* P26E	NZ_LVWI00000000.1	Contig
177	*Paenibacillus humicus* NBRC 102415	NZ_BIMD00000000.1	Contig
178	*Paenibacillus illinoisensis* NBRC 15959	BIME00000000.1	Contig
179	*Paenibacillus jamilae* KACC 10925	QVPU00000000.1	Contig
180	*Paenibacillus kobensis* NBRC 15729	NZ_BILZ00000000.1	Contig
181	*Paenibacillus koleovorans* NBRC 103111	NZ_BIMA00000000.1	Contig
182	*Paenibacillus lactis* DSM 15596	JAGGKI000000000.1	Contig
183	*Paenibacillus lautus* NBRC 15380	BIMF00000000.1	Contig
184	*Paenibacillus lemnae* L7-75	NZ_JABBPN000000000.1	Contig
185	*Paenibacillus lentimorbus* NRRL B-30488	ANAT00000000.1	Contig
186	*Paenibacillus lignilyticus* DLE-14	NZ_JAGKSP000000000.1	Contig
187	*Paenibacillus macerans* NBRC 15307	BIMG00000000.1	Contig
188	*Paenibacillus maysiensis* 1-49	NZ_ASRY00000000.1	Contig
189	*Paenibacillus mendelii* DSM 19248	NZ_JAFBDG000000000.1	Contig
190	*Paenibacillus montanisoli* RA17	NZ_QLUW00000000.1	Contig
191	*Paenibacillus monticola* LC-T2	NZ_WJXB00000000.1	Contig
192	*Paenibacillus nanensis* DSM 22867	NZ_QXQA00000000.1	Contig

Supplementary Information

193	*Paenibacillus naphthalenovorans* PR-N1	FNDY00000000.1	Contig
194	*Paenibacillus nasutitermitis* CGMCC 1.15178	NZ_BMHP00000000.1	Contig
195	*Paenibacillus nicotianae* DSM 28018	NZ_JAFBEN000000000.1	Contig
196	*Paenibacillus nuruki* TI45-13ar	NZ_MDER00000000.1	Contig
197	*Paenibacillus oceani* IB182363	NZ_JACXJA000000000.1	Contig
198	*Paenibacillus ottowii* MS2379	NZ_VIJZ00000000.1	Contig
199	*Paenibacillus pabuli* NBRC 13638	BCNM00000000.1	Contig
200	*Paenibacillus paeoniae* M4BSY-1	NZ_QUBQ00000000.1	Contig
201	*Paenibacillus phocaensis* mt24	FCOQ00000000.1	Contig
202	*Paenibacillus phytohabitans* LMG 31459	NZ_WHOB00000000.1	Contig
203	*Paenibacillus phytorum* LMG 31458	NZ_WHOA00000000.1	Contig
204	*Paenibacillus pini* JCM 16418	BAZT00000000.1	Contig
205	*Paenibacillus pinisoli* JCM 19203	NZ_QXQB00000000.1	Contig
206	*Paenibacillus pinistramenti* ASL46	NZ_VAWG00000000.1	Contig
207	*Paenibacillus piri* MS74	NZ_SMRT00000000.1	Contig
208	*Paenibacillus plantarum* LMG 31461	NZ_WHNY00000000.1	Contig
209	*Paenibacillus planticolens* LMG 31457	NZ_WHNZ00000000.1	Contig
210	*Paenibacillus popilliae* ATCC 14706	NZ_BALG00000000.1	Contig
211	*Paenibacillus rhizophilus* 7197	RQPI00000000.1	Contig
212	*Paenibacillus rhizoplanae* DSM 103963	JAGGLX000000000.1	Contig
213	*Paenibacillus rigui* JCM 16352	NZ_NMQW00000000.1	Contig
214	*Paenibacillus sacheonensis* DSM 23054	JAFBDP000000000.1	Contig
215	*Paenibacillus sambharensis* SMB1	NZ_QKRB00000000.1	Contig
216	*Paenibacillus sediminis* DSM 23491	NZ_JAGGKP000000000.1	Contig
217	*Paenibacillus selenitireducens* ES3-24	NZ_MSZX00000000.1	Contig
218	*Paenibacillus sepulcri* CCM 7311	JAHZIK000000000.1	Contig
219	*Paenibacillus shirakamiensis* DSM 26806	NZ_JAGGLD000000000.1	Contig
220	*Paenibacillus silagei* DSM 101953	NZ_JAGGLV000000000.1	Contig
221	*Paenibacillus taichungensis* DSM 19942	JABMCC000000000.1	Contig
222	*Paenibacillus tepidiphilus* SYSU G01001	VWRQ00000000.1	Contig
223	*Paenibacillus thalictri* N2SHLJ1	SIRE00000000.1	Contig
224	*Paenibacillus timonensis* 12ME58	WNZY00000000.1	Contig
225	*Paenibacillus turicensis* DSM 14349	JAGGKG000000000.1	Contig

226	*Paenibacillus turpanensis* YIM B00363	WTLI00000000.1	Contig
227	*Paenibacillus tyrfis* MSt1	JNVM00000000.1	Contig
228	*Paenibacillus urinalis* DSM 22281	JAFBDL000000000.1	Contig
229	*Paenibacillus validus* NBRC 15382	BIMH00000000.1	Contig
230	*Paenibacillus vortex* V453	ADHJ00000000.1	Contig
231	*Paenibacillus wynnii* DSM 18334	JQCR00000000.1	Contig
232	*Paenibacillus xerothermodurans* ATCC 27380	NHRJ00000000.2	Contig
233	*Paenibacillus xylanexedens* DSM 21292	JAGIKV000000000.1	Contig
234	*Paenibacillus xylaniclasticus* NBRC 106381	BIML00000000.1	Contig
235	*Paenibacillus xylanilyticus* LMG 21957	JABMCB000000000.1	Contig
236	*Paenibacillus zanthoxyli* JH29	ASSD00000000.1	Contig
237	*Paenibacillus zeisoli* 3-5-3	RZNX00000000.1	Contig
238	*Paenibacillus* sp. LS1	JAPDOE000000000.1	Contig

Supplementary Information

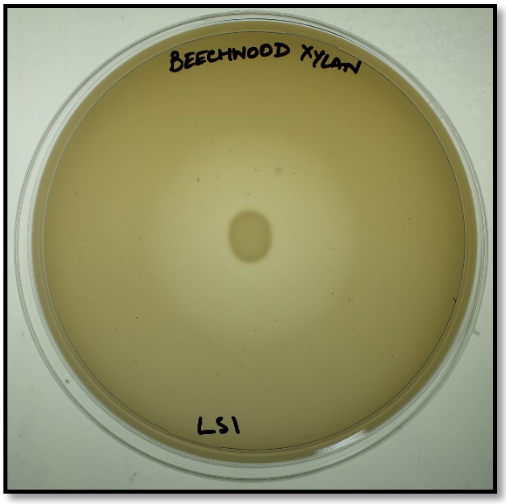

FIG S1: Zone of hydrolysis observed for *Paenibacillus* sp. LS1 spot-inoculated on 1% beechwood xylan agar plate.

Supplementary Information

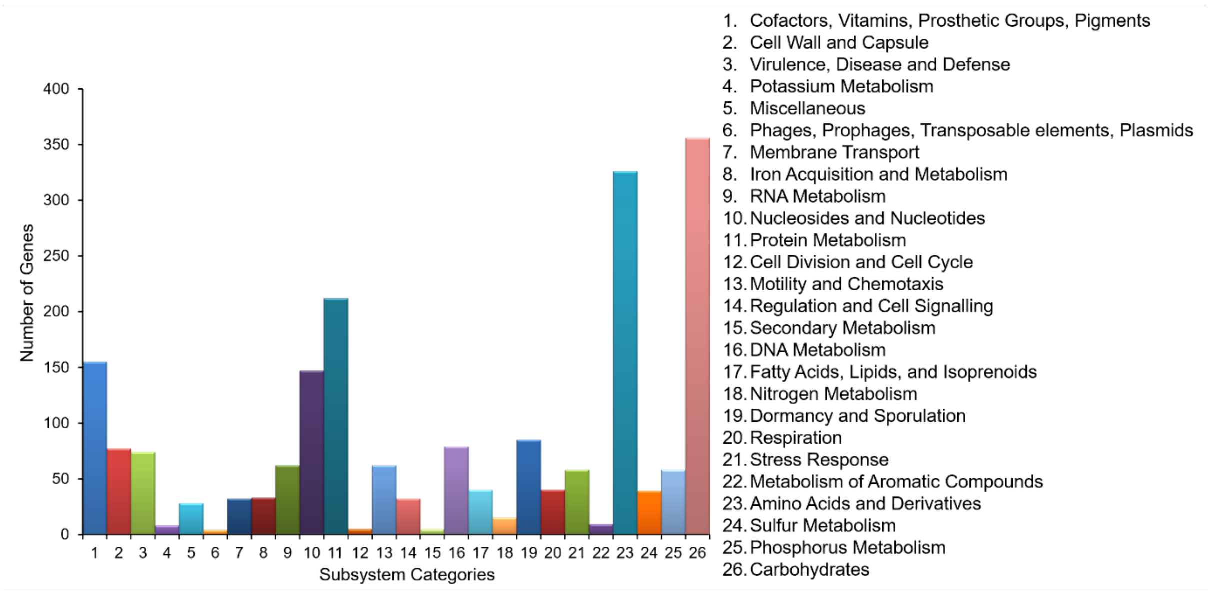

FIG S2: Distribution of genes among 26 subsystem categories in the annotated of genome of *Paenibacillus* sp. LS1 by the Rapid Annotation using Subsystem Technology (RAST) server.

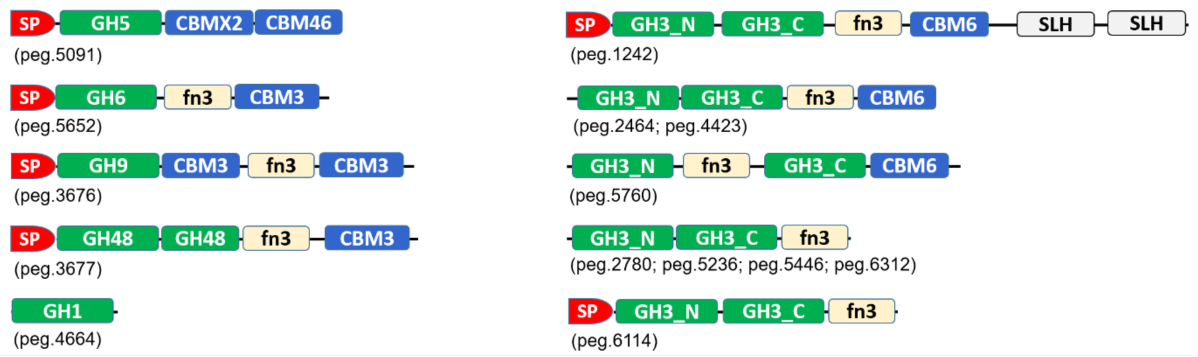

FIG S3: Schematic representation of domain organization of the predicted cellulose degrading CAZymes in *Paenibacillus* sp. LS1. The PATRIC sequence IDs (starting with "peg") of the respective CAZymes with a particular domain architecture are indicated within parenthesis. Abbreviations – SP: Signal Peptide; GH: Glycoside Hydrolase; CBM: Carbohydrate Binding Module; SLH: S-Layer Homology domain; fn3: fibronectin type-3 domain; GH3_N: Glycoside Hydrolase family 3 N-terminal domain; GH3_C: Glycoside Hydrolase family 3 C-terminal domain.
